# Persistent antimicrobial resistance during soil remediation driven by residual heavy metal co-selection

**DOI:** 10.1093/ismejo/wrag058

**Published:** 2026-03-18

**Authors:** Rui Xue, Yiyue Zhang, Hongzhe Li, Jian Li, Wenshun Ke, Shilin Hu, Chaoran Li, Faith Ka Shun Chan, Li Cui

**Affiliations:** State Key Laboratory of Regional and Urban Ecology, Institute of Urban Environment, Chinese Academy of Sciences, Xiamen, Fujian 361021, China; College of Resources and Environment, University of Chinese Academy of Sciences, 19A Yuquan Road, Beijing 100049, China; State Key Laboratory of Regional and Urban Ecology, Institute of Urban Environment, Chinese Academy of Sciences, Xiamen, Fujian 361021, China; State Key Laboratory of Regional and Urban Ecology, Ningbo Observation and Research Station, Institute of Urban Environment, Chinese Academy of Sciences, Xiamen, Zhejiang 361021, China; Zhejiang Key Laboratory of Pollution Control for Port-Petrochemical Industry, CAS Haixi Industrial Technology Innovation Center in Beilun, Ningbo, Zhejiang 315830, China; State Key Laboratory of Regional and Urban Ecology, Research Centre for Eco-Environmental Sciences, Chinese Academy of Sciences, Beijing 100085, China; Ministry of Education Key Laboratory of Ecology and Resource Use of the Mongolian Plateau & Inner Mongolia Key Laboratory of Grassland Ecology, School of Ecology and Environment, Inner Mongolia University, Hohhot, Inner Mongolia 010021, China; College of Environment and Ecology, Hunan Agricultural University, Changsha, Hunan 410128, China; State Key Laboratory of Regional and Urban Ecology, Institute of Urban Environment, Chinese Academy of Sciences, Xiamen, Fujian 361021, China; Department of Environmental Science and Engineering, University of Science and Technology of China, Hefei, Anhui 230026, China; State Key Laboratory of Crop Stress Adaptation and Improvement, Henan University, Kaifeng, Henan 475004, China; School of Geographical Sciences, University of Nottingham Ningbo China, Ningbo, Zhejiang 315100, China; State Key Laboratory of Regional and Urban Ecology, Institute of Urban Environment, Chinese Academy of Sciences, Xiamen, Fujian 361021, China

**Keywords:** heavy metal-contaminated soils, antibiotic resistance, single-cell Raman spectroscopy, co-selection mechanisms, remediation ecology

## Abstract

Remediation of heavy metal-contaminated soil is a global priority, particularly as reclaimed land increasingly intersects with urban development and human exposure. However, the ecological consequences of soil remediation, especially its impact on antimicrobial resistance (AMR) as a global health threat, have remained poorly understood. Here, we combined single-cell Raman–D₂O probing with genome-resolved metagenomics to monitor the dynamics of phenotypic and genotypic resistance to metals and antibiotics during a 120-day remediation of soils with three contamination levels from a lead-zinc smelting site. Although chemical remediation substantially reduced bioavailable metals (by 42%–65%), AMR was not diminished. Instead, both phenotypic activity and gene abundance of metal- and antibiotic-resistant microorganisms increased, resulting in a two- to three-fold increase in AMR-associated health risks. Among 76 metagenome assembled genomes (MAGs) from phenotypic resistance communities, all Cd resistance-associated MAGs harbored multidrug resistance genes, half of which were colocalized with metal resistance determinants, and their prevalence continued to rise with remediation. These findings reveal that although remediation alleviates acute metal toxicity, residual low-concentration bioavailable metals sustain evolutionary selection for resistance, highlighting a disconnect between chemical recovery and biological safety. Moreover, the improved soil nutrient and physiochemical properties of remediated soils further promoted the proliferation of antibiotic-resistant bacteria. This study offers new ecological insights into the unintended consequences of anthropogenic interventions, underscoring the need to integrate biological safety into soil health and safety assessments.

## Graphical Abstract

**Graphical Abstract:**
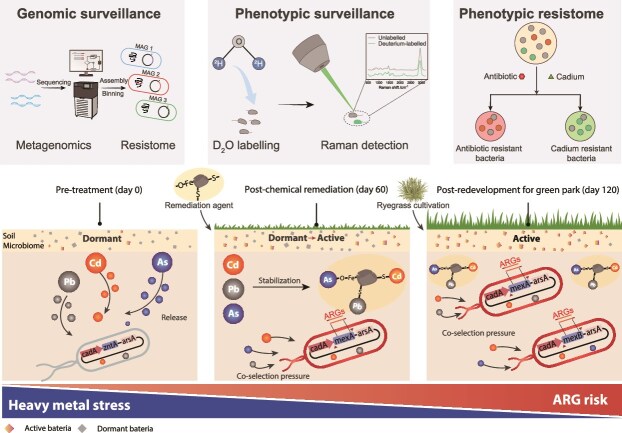

## Introduction

Large-scale heavy metal contamination of soil has become a widespread environmental problem [1]. Remediation of such soil is now a global priority, particularly as contaminated sites are increasingly reclaimed for urban development in response to intense land use in cities [2]. These remediated areas have been frequently repurposed for high-human-contact applications such as residential areas and recreational spaces [2]. Consequently, ensuring the safety of remediated soil is pivotal for protecting human health. Current criteria for the safe use of remediated soils focus primarily on the reduction or removal of chemical pollutants. Modern remediation technologies can substantially lower the bioavailability and toxicity of heavy metals [3, 4]. However, these anthropogenic interventions also reshape soil ecosystems, profoundly influencing microbial communities and their physiological functions [5, 6]. It is therefore important to understand the broader ecological consequences of remediation, particularly the emergence of antibiotic-based antimicrobial resistance (AMR), defined here as bacterial resistance to clinically used antibiotics, as an emerging biological pollutant with global health implications [7].

AMR is a rapidly escalating biological threat capable of disseminating across clinical and environmental settings, posing significant risks to public health [8, 9]. Although improper use of antibiotics is the primary driver, non-antibiotic pollutants also promote resistance through co-selection mechanisms such as co-resistance, co-regulation, and cross-resistance [10]. Heavy metals are one of the most persistent selective pressures for AMR because of their environmental persistence. Field investigations have consistently documented enrichment of antibiotic-resistance genes (ARGs) in metal-contaminated agricultural and mining soils [11–13], with ARG abundance in some sites comparable to those in untreated municipal wastewater [14]. Genomic analyses further revealed direct associations between ARGs and metal-resistance genes (MRGs), highlighting metal-contaminated soils as important reservoirs for resistance evolution and dissemination [15, 16].

To mitigate the ecological and health risks of heavy metals, chemical amendments—such as biochar, and engineered nanomaterials—are widely applied to reduce their bioavailability in soils [17, 18]. Such amendments typically act through adsorption, complexation, or precipitation, reducing the fraction of bioavailable metals down to environmentally safe levels [19]. Despite substantial progress in reducing the bioavailability of heavy metals, the ecological impact of residual low-level metals on shaping soil microbial community and their AMR functions remains largely unexplored and insufficiently assessed, such as maintenance, attenuation, or enhancement. It is important to elucidate such an impact to advance understanding of the ecological risk associated with remediated soils.

Current approaches for monitoring environmental AMR rely primarily on quantitative PCR and metagenomic sequencing that provide comprehensive antibiotic/metal resistance gene inventories. The gene profiles have been widely used for surveillance of environmental AMR, deciphering the driving factor, and even to quantify AMR risks under different conditions [20]. However, genotypic information can only reflect resistance potential but not the actual physiology of microbial populations, such as phenotypic resistance and metabolic activity, which are more directly linked to antibiotic-resistance-related risks arising from microbial activity. Moreover, substantial portions of soil microbial DNA may originate from non-viable cells and extracellular DNA [21], resulting in frequently observed discrepancies between genotypic and phenotypic resistance. It is therefore important to integrate phenotype and genotype for a more tangible understanding of AMR. Single-cell Raman spectroscopy coupled with deuterium oxide (D₂O) labeling offers a culture-independent method for detecting metabolically active resistant bacteria in complex environmental samples [22]. This approach exploits the incorporation of deuterium from D₂O into cellular biomolecules during anabolic metabolism, generating distinctive C–D vibrational signatures detectable by Raman spectroscopy [23]. Under selective pressure from antibiotics or heavy metals, resistant and sensitive cells show differential metabolic activities, enabling quantitative discrimination of metal or antibiotic resistance phenotypes [22, 24]. This method has been successfully applied across diverse ecosystems, including agricultural soils, sediments, host-associated microbiomes, and microplastics [22, 24–26].

Here, the dynamics of AMR in three levels of heavy metal-contaminated soils from a legacy lead–zinc smelting site were investigated over a 120-day remediation and redevelopment. By integrating single-cell Raman–D₂O labeling with metagenomic sequencing, we aimed to (i) comprehensively track temporal dynamics of both phenotype and genotype of antibiotic and metal resistance and their interplay throughout the soil remediation process, (ii) quantify AMR health risk and evaluate their persistence under residual metal concentrations, and (iii) elucidate phenotype-associated microbial community and functional genes to decipher the underlying mechanisms driving the elevated AMR during soil remediation. These investigations provide new ecological insights into how remediation reshapes AMR persistence in contaminated soils.

## Materials and methods

### Site description and experimental design

Soil samples were collected from an abandoned lead-zinc smelting site in Zhuzhou City, Hunan Province, China (27°52′26′′N, 113°04′53′′E), which operated continuously for 60 years until closure and relocation in 2018. The region experiences a subtropical monsoon humid climate with a mean annual temperature of 16°C–18°C. Three soils with varying heavy metal contamination levels were collected and designated as soil L (lightly contaminated), soil M (moderately contaminated), and soil H (heavily contaminated) based on their initial total heavy metal concentrations (Table S1). The soils were ranked and named according to their relative total metal contents.

Soils were air-dried at room temperature for 72 h and passed through a 2-mm sieve to remove stones and plant debris. Remediation was conducted using silica-supported iron sulfide nanomaterials (provided by Henan University) applied at 3% (w/w) to 1000 g of soil in plastic pots. The amendment was sterilized with 70% ethanol for 30 min prior to application. Deionized water was added to maintain a 70% maximum water holding capacity throughout the experiment. After 60 days of chemical remediation, sterilized ryegrass seeds (sterilized with 30% H₂O₂ for 30 min) were planted and cultivated for an additional 60 days under natural light conditions. To realistically simulate the full process of contaminated soil transitioning from remediation to redevelopment as a green space, soil samples were collected at three time points: Day 0 (pre-treatment), Day 60 (post-chemical remediation), and Day 120 (post- redevelopment). Additionally, we included a time-control group consisting of contaminated soils incubated for 120 days without remediation, as well as a material-control group comprising uncontaminated soils (N1, N2) incubated for 120 days with and without the addition of FeS nanoparticles. Control samples were not subjected to metagenomic sequencing. Each sample was divided into three portions for: (i) physicochemical analysis, (ii) single-cell Raman analysis, and (iii) DNA extraction and sequencing.

### Soil physicochemical analysis

Soil pH was measured in a 1:2.5 soil-to-water suspension using a digital pH meter. Available nitrogen was extracted with 1 M KCl and measured colorimetrically. Available phosphorus was extracted using 0.5 M NaHCO₃ (pH 8.5) and determined by the molybdenum blue method.

Bioavailable heavy metals were extracted using DTPA solution (0.005 M diethylenetriamine pentaacetic acid, 0.01 M CaCl₂, 0.1 M triethanolamine, pH 7.3) at a 1:5 soil-to-solution ratio with 2 h shaking at 25°C. Bioavailable arsenic was extracted using 0.5 M NaHCO₃. Heavy metal leaching toxicity primarily reflects the potential risk of heavy metals being mobilized through rainfall leaching or water-mediated transport in soils, which were determined by water extraction at a solid-to-liquid ratio of 1:10, followed by shaking for 8 h at 25°C. Metal concentrations were determined by inductively coupled plasma optical emission spectrometry (ICP-OES, Agilent 700 series).

### Single-cell Raman–D₂O labeling optimization

Standard bacterial strains with known Cd resistance (*n* = 2) and sensitivity (*n* = 2) were used to optimize labeling conditions. Strains were inoculated into sterilized soil H and incubated with D₂O (99 atom% D, Sigma–Aldrich) to determine optimal labeling duration. Time-course experiments (0, 12, 24, 48, 72 h) were conducted with 20% D₂O (v/w) at 25°C.

For Cd concentration optimization, sterilized soils were inoculated with resistant and sensitive strains and treated with 0, 130, 260, and 390 ppm Cd^2+^ (provided as CdCl₂·2.5H₂O) along with D₂O labeling. Soil microorganisms were extracted using Nycodenz density gradient centrifugation: 1 g of soil was mixed with 5 ml PBS containing 0.02% Tween 20, vortexed for 30 min, layered over 5 ml of 0.8 g ml^−1^ Nycodenz solution, and centrifuged at 10 000 × *g* for 60 min. The intermediate layer was collected and washed three times with deionized water.

### Single-cell Raman spectroscopy for phenotype detection

Fresh soil samples were air-dried for 24 h before analysis. Four phenotypic traits were examined: (i) native metabolic activity, (ii) Cd resistance, (iii) ciprofloxacin resistance, and (iv) cefotaxime resistance. For each phenotype, 1 g of soil was amended with 200 μL D₂O containing: (i) 10 mM glucose (control), (ii) 10 mM glucose +130 ppm Cd, (iii) 10 mM glucose +400 ppm ciprofloxacin hydrochloride, or (iv) 10 mM glucose +640 ppm cefotaxime. The use of the Raman–D₂O method to assess the activity of antibiotic-resistant bacteria in soil was originally developed by previous studies [22], following this established framework, our study applied the same approach. All solutions were filter-sterilized (0.22 μm) immediately before use.

Samples were incubated at 25°C for 48 h, followed by Nycodenz density gradient extraction. Single-cell Raman spectra were acquired using a LabRAM Aramis confocal Raman microscope (HORIBA Jobin-Yvon) with a 532-nm Nd: YAG laser and 300 grooves/mm grating. Spectra were collected using a 100× objective (Olympus) with 15 s integration time across 500–3200 cm^−1^. Deuterium incorporation was quantified using the C–D/(C–D + C–H) ratio, calculated from integrated peak areas at 2040–2300 cm^−1^ (C–D) and 2800–3100 cm^−1^ (C–H). A minimum of 120 spectra were collected per sample, with the threshold for metabolic activity set at the mean + 3SD of non-labeled controls.

### DNA extraction and metagenomic sequencing

Total DNA was extracted from 0.5 g of soil using the FastDNA Spin Kit for Soil (MP Biomedicals) following the manufacturer’s protocols. DNA quality was assessed by agarose gel electrophoresis and quantified using a NanoDrop spectrophotometer. Absolute abundance of 16S rRNA genes was determined by qPCR using SYBR Green on a LightCycler 480 system (Roche) with triplicate technical replicates. The forward and reverse primers used were 5ose gel electrophoresis and quantified using a NanoDrop spectrophotometer. Absolute abundance of 16S rRNA genes was determined [27]. Metagenomic libraries were prepared and sequenced on a DNBSEQ-T7 platform (Wefind Biotechnology, Wuhan), generating 150-bp paired-end reads. Raw data have been deposited in the NCBI Sequence Read Archive under accession number PRJNA1332155 and are publicly available. Quality control was performed using Fastp v0.20.0 with parameters: minimum length 50 bp, quality score > Q20, and adapter removal. High-quality reads were assembled using MEGAHIT v1.2.9 with k-mer sizes from 21 to 121 (step size 10).

### Metagenome-assembled genome construction and annotation

Contigs ≥1000 bp were binned using vamb v1.0.0. Metagenome-assembled genomes (MAGs) were quality-filtered using CheckM v1.2.0, retaining only MAGs with ≥90% completeness and ≤10% contamination. Taxonomic classification was performed using GTDB-Tk v2.3.0 with the GTDB R214 database. Open reading frames were predicted using Prodigal v2.6.3. Gene annotation was conducted using DIAMOND (v0.8.35) against multiple databases: SARG 2.0 (antibiotic resistance genes), BacMet v2.0 (metal resistance genes), VFDB (virulence factors), and custom MGE databases (mobile genetic elements), retaining hits with $\ge$80% amino-acid identity and an e-value $\le$ 1 × 10^−5^. ARG profiling was performed using ARGs-OAP (v3.2) with default parameters. Alignments required >75 nucleotides, E-value <1 × 10^−7^, and >80% identity for ARG/MRG assignment [28].

### Antibiotic-resistance gene and metal-resistance gene co-occurrence analysis

MAG-based ARG–MRG co-occurrence analyses were conducted using contigs >1000 bp simultaneously harboring ≥1 ARG and ≥ 1 MRG, which were defined as ARG–MRG co-carrying MAGs. In addition, within the ARG–MRG co-carrying MAGs, those in which the ARG and MRG were located within 5 kb on the same contig were classified as having potential for co-selection. The types of ARGs and MRGs, their genomic distances, and their contig origins were recorded (Table S2).

### Health risk assessment of antibiotic-resistance genes

The health risk of soil ARGs was comprehensively evaluated by integrating multiple factors, including ARG abundance, human accessibility, mobility, human pathogenicity, clinical relevance, and microbial activity [29]. Initially, the inherent risk level of each ARG type was determined based on its potential for human exposure, mobility, association with human pathogens, and clinical importance, following criteria established in previous studies. Subsequently, a risk index was calculated for each ARG type as follows:


(1) \begin{equation*} \mathrm{R}{\mathrm{I}}_{\mathrm{ARG}}=\mathrm{HA}\times \mathrm{MO}\times \mathrm{HP}\times \mathrm{CP} \end{equation*}

where HA = human accessibility, MO = mobility, HP = human pathogenicity, and CA = clinical availability, scored 1–4 based on established criteria.

Sample-level risk indices were calculated as:


(2) \begin{equation*} \mathrm{R}{\mathrm{I}}_{\mathrm{sample}}=\sum_{\mathrm{i}=1}^{\mathrm{n}}{\mathrm{Abundance}}_{\mathrm{i}}\times{\mathrm{RI}}_{\mathrm{i}} \end{equation*}

where ${\mathrm{Abundance}}_{\mathrm{i}}$ is the abundance of ARG in the sample, and ${\mathrm{RI}}_{\mathrm{i}}$ is the RI of ${\mathrm{ARG}}_{\mathrm{i}}$ calculated by eq 1.

Second, microbial metabolic activity was incorporated into the assessment, as elevated activity is closely associated with an increased likelihood of ARG dissemination through cell proliferation or horizontal gene transfer (HGT) [30]. The activity-adjusted risk index was calculated as follows:


(3) \begin{equation*} \mathrm{R}{\mathrm{I}}_{\mathrm{sample}}^{\ast }={\mathrm{RI}}_{\mathrm{j}}\times \mathrm{CImp}{\mathrm{ratio}}_{\mathrm{j}}\times{\mathrm{P}}_{\mathrm{j}} \end{equation*}

where ${\mathrm{RI}}_{\mathrm{j}}$ is the RI of sample_j_ calculated by eq 2, $\mathrm{C}\hbox{--} \mathrm{D}\ {\mathrm{ratio}}_{\mathrm{j}}$ is the whole activity of ${\mathrm{sample}}_{\mathrm{j}}$, based on Raman spectroscopy, and ${\mathrm{P}}_{\mathrm{j}}$ is the proportion of active microbes in ${\mathrm{sample}}_{\mathrm{j}}$.

### Statistical analysis

Statistical analyses were performed using R v4.4.1. Normality was assessed using Shapiro–Wilk tests. One-way ANOVA with Tukey’s HSD *post hoc* test was used for multiple comparisons. Permutational multivariate analysis of variance (PERMANOVA) was conducted using the vegan package with 9999 permutations. Spearman correlation analysis was performed to assess relationships between resistance phenotypes and MAG abundances using the psych package. Principal coordinates analysis (PCoA) was based on Bray–Curtis dissimilarity matrices. Linear discriminant analysis effect size (LEfSe) was used to identify stage-specific ARG enrichment patterns with LDA score > 2.0. Correlations between resistance phenotypes and environmental parameters were assessed using Spearman’s rank correlation with significance set at α < 0.05. All visualizations were generated using ggplot2 v3.3.5.

## Results

### Remediation effectively reduces bioavailable heavy metals and alters soil microbial communities

**Figure 1:**
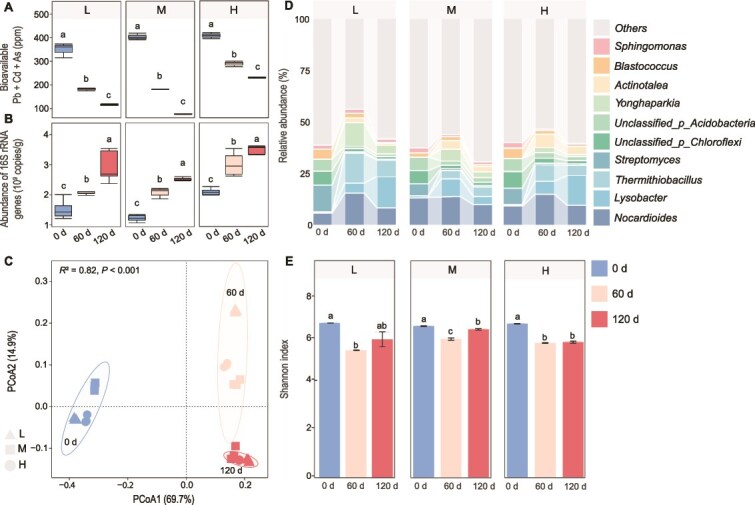
Remediation efficiency and microbial community responses in heavy metal-contaminated soils. (A) Bioavailable heavy metal concentrations (Pb, Cd, As) across remediation timeline in soils with varying contamination levels (L, lightly contaminated; M, moderately contaminated; H, heavily contaminated). (B) Bacterial biomass dynamics estimated by absolute abundance of 16S rRNA genes. (C) PCoA of bacterial community composition based on Bray–Curtis dissimilarity. Ellipses denote 95% confidence intervals grouped by sampling time (blue, Day 0; orange, Day 60; red, Day 120). Shapes represent contamination levels (triangle, L; square, M; circle, H). (D) Temporal dynamics of dominant bacterial genera. Taxa are ordered by their relative abundance across remediation treatments. (E) Alpha diversity (Shannon index) changes during remediation. For (A), (B), and (E), different letters indicate statistically significant differences among time points within each soil type (one-way ANOVA with Tukey’s HSD, *P* < .05). All data are based on three biological replicates.

To quantitatively evaluate remediation effectiveness, we monitored changes in bioavailable heavy metals in three soils with different contamination levels over the 0-, 60-, and 120-day remediation period. The nano-sized iron sulfide remediation treatment substantially reduced bioavailable heavy metals across all three levels of contaminated soils (Fig. 1A, Fig. S1). After 60 days of treatment, bioavailable Pb, Cd, and As concentrations decreased by 42.94%, 64.89%, and 34.47%, respectively. During the ryegrass cultivation stage (60–120 days), bioavailable metals continued to decline. Bioavailable Pb consistently decreased across all soil types, whereas As declined only in the low-contamination soil (L) (Fig. S1). Throughout the remediation period, the leaching toxicity heavy metal concentrations in all treated soils reached a level of Pb < 0.05, Cd < 0.02, and As <0.005 mg l^−1^, all meeting China’s GB3838-2002 standard for regulatory acceptance criteria for contaminated site remediation (Fig. S2). This demonstrates that all the heavy metal-contaminated soil has been remediated to environmentally acceptable levels.

In parallel with metal dynamics, we further characterized the temporal shifts in soil microbiomes to elucidate how microbial communities responded to remediation across the same time points. Quantitative analysis of 16S rRNA genes revealed progressive bacterial abundance increases from Day 0 to Day 120, with copy numbers rising by 97.35% from 1.51 × 10^9^ to 2.98 × 10^9^ copies g^−1^ in soil L (Fig. 1B, Fig. S3). Similar trends occurred in moderately (soil M, 103.15% increase) and heavily contaminated soils (soil H, 68.84% increase), indicating that bacterial biomass significantly increased with remediation. PCoA revealed significant temporal shifts in community composition (Fig. 1C), indicating that the remediation process substantially restructured bacterial communities. The microbial community was dominated by *Nocardioides*, *Streptomyces*, and *Blastococcus* in unremediated soils (Fig. 1D), but shifted to *Lysobacter*, *Thermithiobacillus*, and *Actinotalea* (Fig. 1D) after remediation. Shannon diversity indices declined following remediation and recovered after ryegrass growth (Fig. 1E), indicating that chemical remediation alone negatively impacted microbial diversity.

### Single-cell Raman–D₂O labeling enables quantification of phenotypically active Cd-resistant bacteria in soil

Given that Cd exhibits the highest mobility among heavy metals in contaminated soils due to its weaker binding affinity to soil particles [31, 32], it presents the greatest potential for co-selection of ARGs through spatial dissemination. Therefore, *in situ* metabolically active Cd-resistant bacteria were studied by a single-cell Raman–D₂O approach.

**Figure 2:**
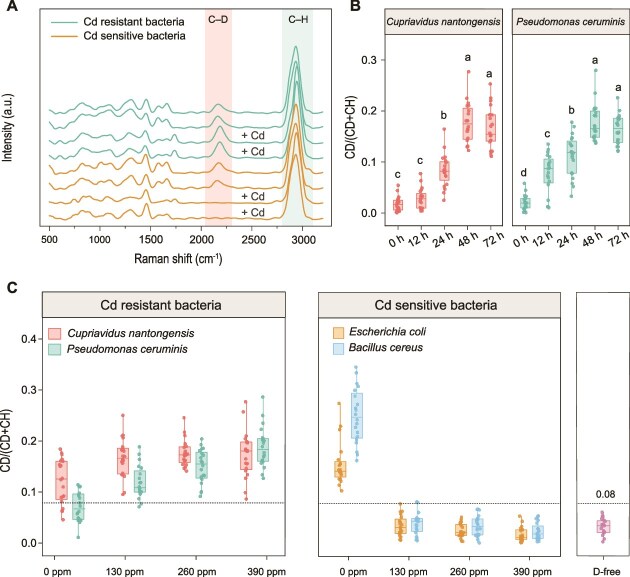
Development and validation of single-cell Raman–D₂O method for detecting active cadmium-resistant bacteria. (A) Representative single-cell Raman spectra of cadmium-resistant and cadmium-sensitive bacterial strains incubated with D₂O in the presence or absence of Cd^2+^ stress. Shaded regions highlight C–D (2040–2300 cm^−1^) and C–H (2800–3100 cm^−1^) vibrational bands used for activity quantification. (B) Time-course optimization of D₂O labeling efficiency in cadmium-resistant bacteria inoculated in heavily contaminated soil. Different letters indicate statistically significant differences among time points (one-way ANOVA with Tukey’s HSD, *P* < .05). C–D ratios calculated as CD/(CD + CH) based on integrated band areas. Data points represent individual cells (*n* = 20 per time point). The optimal labeling duration (48 h) was determined based on signal plateau analysis. (C) Concentration-dependent discrimination of cadmium-resistant versus cadmium-sensitive bacteria under varying Cd^2+^ concentrations. Horizontal dashed line indicates the detection threshold (C–D ratio = 0.08) determined as mean + 3SD of unlabeled controls.

To establish the methodological foundation for phenotypic resistance detection, we first optimized Raman–D₂O labeling conditions using pure cultures. Two Cd-resistant strains maintained robust metabolic activity and growth in both Cd-containing (+ Cd) and Cd-free media, evidenced by strong C–D spectral signals (2040–2300 cm^−1^), whereas Cd-sensitive strains showed C–D signals and growth only under Cd-free conditions (Fig. 2A, Fig. S4).

Time-course experiments using two Cd-resistant bacteria inoculated into sterilized highly contaminated soil demonstrated that D₂O incorporation peaked at 48 h, with C–D ratios plateauing at this time point (Fig. 2B). Considering that the bioavailable heavy metal in soil is decreasing during remediation, and the heavy metal in the original soil is not enough to differentiate Cd-sensitive and Cd-resistant bacteria (Fig. S1), additional Cd was added, and the dosage was evaluated. Dose–response experiments by inoculating two resistant and two sensitive strains into sterilized soils amended with 0, 130, 260, and 390 ppm Cd^2+^ were performed. The result established 130 ppm Cd^2+^ as the concentration for distinguishing Cd-resistant from Cd-sensitive bacteria in soil matrices. At this concentration, Cd-resistant strains maintained high metabolic activity across all tested Cd gradients, whereas sensitive strains exhibited complete activity suppression at 130–390 ppm Cd^2+^ (Fig. 2C). This concentration thus provided sufficient bioavailable Cd to enable reliable phenotypically Cd-resistance and sensitive microbe discrimination.

### Remediation enhances active antibiotic-resistant bacteria despite metal reduction

**Figure 3:**
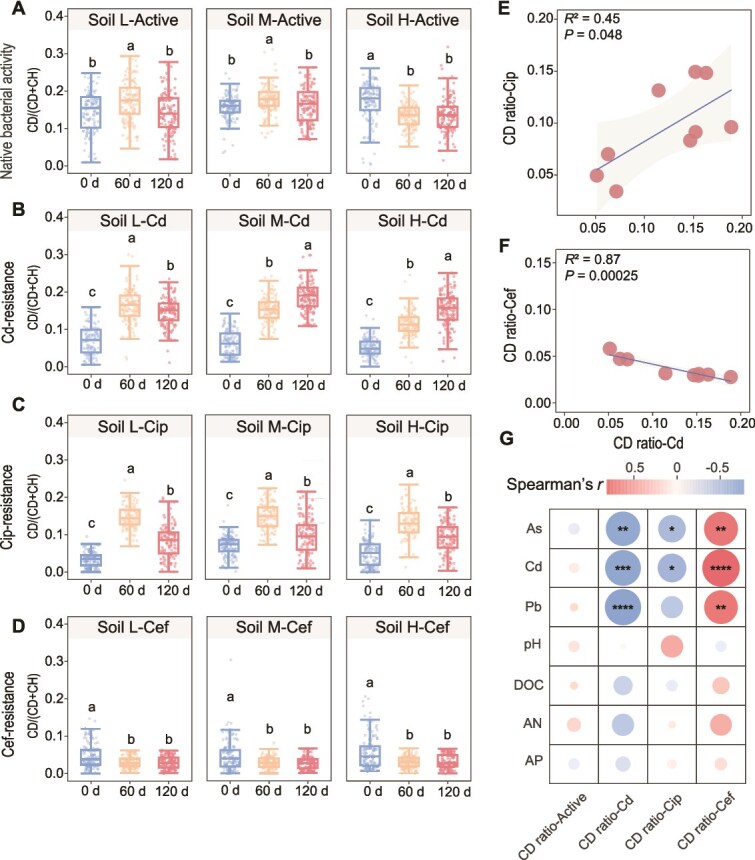
Phenotypic activity dynamics of distinct bacterial populations during soil remediation. (A–D) Metabolic activity profiles of four bacterial phenotypes: (A) native active bacteria, (B) cadmium-resistant bacteria, (C) ciprofloxacin-resistant bacteria, and (D) cefotaxime-resistant bacteria. Activity quantified using C–D ratios from single-cell Raman spectroscopy following D₂O labeling. Different letters denote significant differences among time points within each soil type (*P* < .05, one-way ANOVA with Tukey’s HSD). (E) Spearman correlation analysis between cadmium-resistant and ciprofloxacin-resistant bacterial activities across all samples. Each point represents an individual sample colored by time point. The blue line shows the linear regression fit with 95% confidence intervals (gray shading), indicating a significant positive correlation (*R*^2^ = 0.45, *P* < .05). (F) Spearman correlation analysis between cadmium-resistant and cefotaxime -resistant bacterial activities across all samples. The blue line shows the linear regression fit with 95% confidence intervals (gray shading), indicating a significant negative correlation (R^2^ = 0.87, *P* < .001). (G) Spearman correlation heatmap between bacterial phenotypic activities and soil physicochemical parameters, with As, Cd, and Pb referring to their bioavailable fractions. Color intensity represents correlation strength; asterisks indicate significance levels (^*^P < .05, ^**^P < .01, ^***^P < .001).

Using the optimized Raman–D₂O approach, we tracked dynamic shifts of active bacterial communities with four specific functions throughout the remediation of three levels of polluted soil: native bacterial activity, Cd resistance, and antibiotic resistance to ciprofloxacin and cefotaxime. Native bacterial activity showed variable temporal responses without a consistent trend in low, moderate, and heavy-contamination soil (Fig. 3A). By comparison, Cd-resistant bacterial activity increased consistently throughout the 120 days of remediation in nearly all soil types (Fig. 3B), indicating that Cd-resistant bacteria survive well in the mitigated Cd stress. In addition, ciprofloxacin-resistant bacterial activity increased significantly at Day 60 and then declined at Day 120. Nevertheless, activity at Day 120 remained significantly higher than at Day 0 (Fig. 3C). A significant positive correlation was observed between Cd-resistant and ciprofloxacin-resistant bacterial activities (Fig. 3E), suggesting a potential co-occurrence of metal and antibiotic resistance. In contrast, cefotaxime-resistant bacterial activity declined precipitously and became undetectable after remediation (Fig. 3D). Additionally, Cd-resistant and cefotaxime-resistant bacterial activities were negatively correlated (Fig. 3F), suggesting that competitive community succession during remediation may have contributed to the progressive loss of cefotaxime-resistant activity. In addition, no significant differences were observed among the four phenotypic categories in the unremediated temporal control compared with Day 0, indicating that the observed microbial phenotypic changes were driven by remediation rather than by other confounding factors (Fig. S5). To exclude false-positive phenotypes arising from sorption of heavy metals or antibiotics by remediation materials, we inoculated sterilized soils with two strains known to be sensitive to both stressors and monitored phenotypic activity under metal or antibiotic addition. Neither strain exhibited activity in any treatment, confirming the robustness of our soil phenotypic assays for heavy-metal and antibiotic resistance (Figs S6–S7). Additionally, to rule out the possibility that FeS nanoparticles might alter microbial antibiotic resistance through potential cytotoxic or stress effects, we evaluated antibiotic resistance in two uncontaminated soils after 120 days of incubation with and without FeS addition. The results showed that FeS treatment had no significant impact on antibiotic resistance—either ciprofloxacin or cefotaxime resistance—in either soil type (Fig. S8).

Correlation analysis with soil parameters revealed that bioavailable Pb and Cd were negatively correlated with both Cd- and ciprofloxacin-resistant activities, indicating the persistent and even enhanced effect of metal residues on phenotypic ciprofloxacin resistance, whereas their positive correlation with cefotaxime-resistant activity (Fig. 3G) suggests a selective effect of heavy metals on phenotypic antibiotic resistance.

### Remediation alters soil resistome composition and elevates health risks of antimicrobial resistance

**Figure 4:**
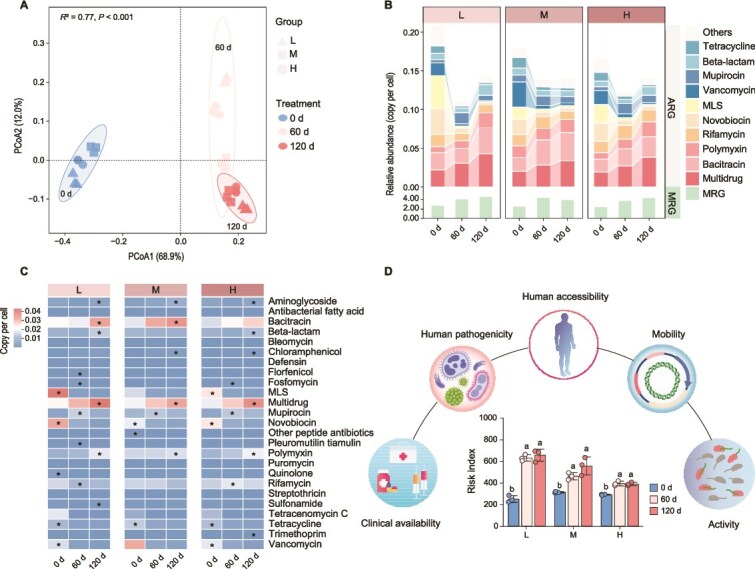
Antibiotic resistance gene dynamics and health risk assessment during remediation. (A) PCoA of antibiotic resistome composition based on Bray–Curtis dissimilarity. Trajectory analysis reveals significant temporal shifts in resistance gene profiles (PERMANOVA: *R*^2^ = 0.77, *P* < .001). (B) Temporal profiles of ARG and MRG abundances normalized to copy number per cell. Stacked bar charts show ARG composition by functional classes; line graphs display total MRG abundance trends. (C) Linear discriminant analysis (LDA) effect size (LEfSe) identification of time-point enriched ARGs across contamination levels. The heat map displays the relative abundance of resistance genes, with significantly enriched genes indicated by asterisks (LDA > 2.0, *P* < .05). Color intensity represents enrichment magnitude from low (blue) to high (red). (D) Integrated health risk assessment of soil AMR incorporating abundance, mobility, pathogenicity, clinical relevance, and bacterial activity. Risk indices calculated using established weighting factors and normalized to baseline values.

Metagenomic analysis further revealed the temporal shifts of ARG and MRG profiles in all three soils during remediation. PCoA confirmed significant temporal changes in resistome composition during remediation (Fig. 4A). The relative abundance of MRGs generally increased throughout the remediation in all three soils, consistent with the increase of phenotypic activity of Cd-resistant bacteria and the overall microbial biomass (Fig. 4B, Fig. 3B). The relative abundance of ARGs was found to decline after 60 days and then slightly recover after 120-day remediation. Among them, multidrug, bacitracin, polymyxin, rifamycin, and β-lactam resistance genes dominated across all soil samples and remediation processes (Fig. 4B). The relative abundances of multidrug, aminoglycoside, and polymyxin resistance genes increased consistently throughout the remediation period across all soil types, whereas the relative abundances of resistance genes associated with novobiocin, tetracycline, vancomycin, tetracenomycin C, MLS, and other peptide antibiotics decreased during remediation (Fig. 4C, Table S3).

A further linear discriminant analysis identified stage-specific ARG enrichment patterns (Fig. 4C). Novobiocin and tetracycline resistance genes were broadly enriched across all samples at Day 0. By Day 60, mupirocin resistance genes were enriched in all samples. Upon completion of remediation (Day 120), aminoglycoside, multidrug, and polymyxin resistance genes enrichment was universal across all samples, indicating a shift in the enriched ARG profiles over the remediation period.

In addition, we specifically examined the dynamics of ARGs associated with two phenotypic AMR. Quinolone resistance genes consistently declined across all soil types throughout the entire remediation process (Fig. S9). Cefotaxime-specific resistance genes, such as *blaCTX-M*, *blaTEM*, and *blaSHV* [33], were virtually undetectable in all samples, except for a few occurrences in the M soil (Table S4). Obviously, the genotypic changes were not consistent with the phenotypic change of both ciprofloxacin and cefotaxime-resistant bacteria, indicating the importance of incorporating both for analysis.

Given that remediated sites are often repurposed for human use, such as parks or green spaces, assessing AMR-related health risks during remediation is critical for safeguarding public health. By using both phenotypic and genotypic AMR (Table S5), including abundance, human accessibility, mobility, human pathogenicity, clinical availability, mobility, pathogenicity, activity, a more realistic assessment of the health risk of AMR in all three soils during remediation were conducted [29].

Despite successful metal immobilization, ARG-associated health risks increased significantly during the first 60 days of remediation, then stabilized through Day 120 (Fig. 4D), indicating an enhanced and persistent AMR risk even after soil remediation. Moreover, risk indices varied considerably among contamination levels, with lightly contaminated soil exhibiting the highest post-remediation ARG risks, followed by moderately and heavily contaminated soils. These findings clearly highlight that AMR risks in the remediated soil remain a serious concern, even in low-contamination scenarios.

### Phenotype-associated genomics analysis reveals driving mechanisms of antimicrobial resistance under metal reduction

**Figure 5:**
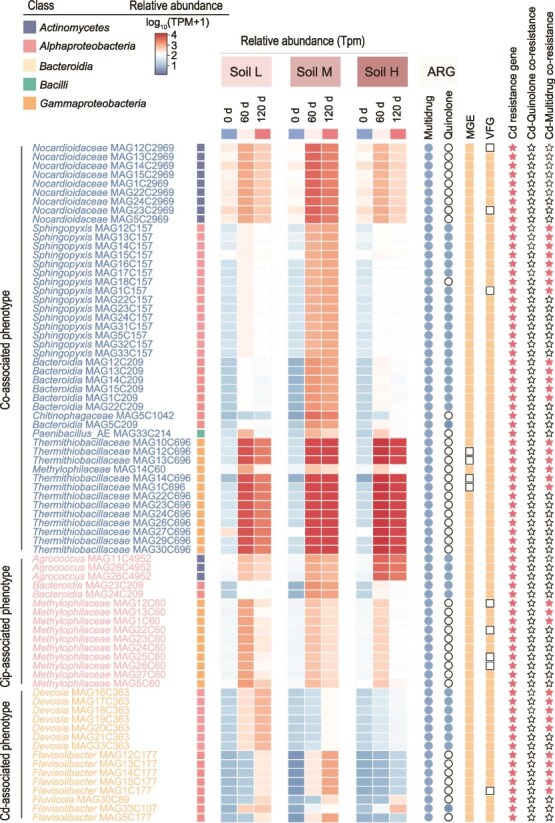
Genomic basis of phenotype-associated bacterial populations and co-selection mechanisms. Phylogenetic analysis and functional annotation of MAGs significantly correlated with cadmium resistance and ciprofloxacin resistance phenotypes (Spearman’s *r* > 0.6, *P* < .05). Branch colors represent taxonomic classes. Right panel: Heat map showing relative abundance (transcripts per million, TPM) of selected MAGs across soil types and time points. Annotation tracks (from left to right) indicate the taxonomic affiliation of each MAG, changes in its relative abundance during remediation in the L, M, and H soils, and the presence of ARGs, MGEs, VFGs, and Cd-resistance genes. Stars indicate MAGs harboring ARG-MRG co-existed genomic fragments.

To elucidate the genetic foundations underlying the observed phenotypic patterns, we analyzed the correlations between metabolic activities of resistance phenotypes and abundance of MAGs. From 600 high-quality MAGs (≥ 90% completeness, ≤ 10% contamination), 60 exhibited significant positive correlations with ciprofloxacin resistance and 62 with Cd resistance (Fig. 5). Specifically, ~60% of the phenotype-associated MAGs (46 out of 76) were concurrently correlated with both ciprofloxacin and Cd resistance traits. Five bacterial classes—*Actinomycetes*, *Alphaproteobacteria*, *Bacteroidia*, *Bacilli*, and *Gammaproteobacteria*—were the predominant taxa among phenotype-associated MAGs. Critically, all MAGs associated with a Cd resistance phenotype not only carried Cd resistance genes but also harbored multidrug resistance genes, indicating that Cd-resistant bacteria serve as important reservoirs of ARGs (Fig. 5, Table S6). Consistently, two Cd-resistant isolates obtained from the remediated soil were also found to carry multidrug resistance genes, further supporting this conclusion (Fig. S10, Table S7). Furthermore, all MAGs associated with ciprofloxacin resistance also carried multidrug and Cd resistance genes, whereas fewer than 10% contained quinolone resistance genes. This pattern indicates that ciprofloxacin-resistant MAGs more frequently co-occurred with multidrug ARGs than with quinolone ARGs.

Genetic linkage analysis revealed that ~42% (32/76) of the MAGs from phenotypic resistance communities exhibited co-resistance potential for Cd and multidrug resistance, with Cd resistance genes and multidrug resistance genes located on the same contig within a distance of ≤5 kb (Table S2). In contrast, none of the MAGs showed Cd–quinolone co-resistance potential, indicating that cadmium exerts a stronger co-resistance preference for multidrug resistance genes than for quinolone resistance genes. In addition, among the phenotype-associated MAGs that became highly enriched after remediation, several MAGs belonging to the Thermithiobacillaceae family (e.g. MAG1C696, MAG10C696, MAG12C696, MAG13C696, and MAG14C696) contained Cd resistance genes and multidrug resistance genes on the same contig (Table S2). This indicates that certain taxa with co-resistance potential were indeed enriched following remediation. In addition, several low-abundance phenotype-associated MAGs (e.g. MAG1C60 and MAG12C60) carried MRGs, ARGs, and transposase genes on the same contig, with the distance between transposase genes and MRGs being ≤10 kb (Table S8). This genomic configuration may indicate a potential for MGEs-mediated co-dissemination of ARGs and MRGs. Contigs co-carrying Cd resistance and multidrug resistance genes increased consistently throughout remediation in nearly all samples (Fig. S11). Similarly, contigs co-carrying Cd resistance and quinolone resistance genes showed comparable patterns except significant declines in heavily contaminated soil, indicating the persistent co-selection pressure exerted by heavy metals on ARGs throughout the remediation process. Moreover, over 80% (64/76) of these MAGs also encoded virulence factors genes (VFGs) and MGEs, suggesting the potential risk for HGT pathogenicity in soil environments.

## Discussion

This study shows that successful chemical remediation of heavy metal-contaminated soil does not eliminate AMR risks, but may enhance them through persistent co-selection mechanisms. Through combined phenotypic and genotypic profiling of heavy metal and antibiotic resistance, we observed that a 42%–65% reduction in bioavailable metals was accompanied by a two to three-fold increase in AMR-associated health risks, highlighting a biological safety issue that warrants further attention. Based on phenotypic resistance-associated MAGs by linking single-cell phenotype with metagenomic sequencing, we show that residual metals maintain sufficient selective pressure to drive antibiotic resistance gene enrichment through co-resistance, challenging current assumptions about the biological safety of remediated environments.

### Single-cell approaches complement metagenomic analysis for a comprehensive resistome assessment

The integration of single-cell phenotypic measurements with metagenomic analysis provides complementary perspectives on environmental resistomes that neither approach alone can achieve. Metagenomic sequencing excels at providing comprehensive metal/antibiotic resistance gene inventories across entire communities [34], whereas the presence of a resistance gene does not necessarily confer a detectable resistance phenotype. Recent findings in environmental microbial communities under varying stress conditions [25] demonstrated that no corresponding resistance phenotype was observed for up to 40% of the detected resistance genes. Moreover, substantial portions of soil microbial DNA were found to originate from non-viable sources [21].

By comparison, the single-cell Raman–D₂O approach adds critical insights into the resistance expression patterns and metabolic activity states against heavy metals and antibiotics. The Raman–D₂O approach employs the intensity of the C–D band (≈ 2040–2300 cm^−1^) as a quantitative indicator of bacterial anabolic activity under heavy-metal or antibiotic stress. Resistant bacteria are able to maintain anabolic activity when exposed to these stressors, enabling their phenotypic discrimination from sensitive populations [35]. Both Cd- and ciprofloxacin-resistance were revealed to increase with remediation, and their phenotype changes were more synchronous with the increase of multidrug ARGs and metal resistance genes, but not with quinolone ARGs. In fact, ciprofloxacin resistance is predominantly driven by mutations in the drug targets, topoisomerase IV and DNA gyrase, whereas mobile quinolone resistance genes contribute only marginally [36, 37], which may explain the weak correspondence between quinolone ARG abundance and Raman-based phenotypic resistance.

Similar disconnect was observed between β-lactam gene and cefotaxime resistance activity. These findings illustrate the importance of introducing phenotypes to complement the conventional genotype-based AMR profile and risk evaluation. In our work, we comprehensively integrated both genotypic and phenotypic parameters, including human accessibility, mobility, human pathogenicity, clinical availability, abundance of ARGs (genotype), and bacterial resistance activity (phenotype), to thoroughly evaluate antibiotic resistance risks throughout the remediation process of contaminated soils. Although the overall relative abundance of ARGs shows a declining trend, consistent with previous studies demonstrating that phytostabilization effectively reduces ARG abundance in copper-contaminated tailings [38], the risk of AMR was found to increase by two to three fold. Such a discrepancy indicated that introducing phenotypic parameters to reflect actual resistance expression may lead to more tangible underestimation of resistance risks at remediated sites.

Linkage of phenotype and genotype also enables us to target phenotype-associated MAGs. From these MAGs, the main taxa and functional genes associated with the elevated biological risk were revealed. For example, microbes exhibiting ciprofloxacin resistance phenotypes were found to predominantly harbor multidrug resistance genes, instead of quinolone-specific resistance genes.

Additionally, a progressive enrichment of multidrug resistance genes over the course of remediation closely mirrored the trend in ciprofloxacin resistance phenotypes. These findings suggest that the observed ciprofloxacin resistance should be largely driven by the expression of multidrug resistance mechanisms. Therefore, the ability to track both resistance phenotypes and genotypes in complex soil matrices throughout remediation represents an important methodological advance for environmental resistance monitoring.

### Elevated antibiotic-resistance gene health risks persist despite successful remediation of heavy metal-contaminated soil

The consistent increase in multidrug resistance genes and metabolic activity of ciprofloxacin-resistance bacteria across all soil types during remediation, despite substantial metal reduction, indicates that biosafety concerns may still persist after remediation and require further attention. The further risk assessment of AMR via an integrated phenotype and genotype information revealed that activity-adjusted risk indices increased by two to three fold, despite effective immobilization of heavy metals and decrease of relative abundance of ARGs. This elevated risk was largely driven by the co-selection pressure exerted by residual bioavailable heavy metals after remediation and by the enrichment of certain resistant taxa during the remediation process.

Metal-resistant bacteria (MRB), which possess adaptive traits that enable survival in metal-contaminated environments [39], are frequently isolated from polluted sites and applied in the bioremediation of contaminated soils and wastewater [40–42], and are therefore often regarded as beneficial microorganisms. However, our study revealed that MRB may pose unrecognized risks. We found that bacteria exhibiting Cd resistance phenotypes commonly carried multidrug resistance genes, and the metabolic activity of Cd-resistant bacteria was significantly positively correlated with that of ciprofloxacin-resistant bacteria. These findings suggest that the proliferation of Cd-resistant bacteria during remediation may play a key role in driving the increased expression of ciprofloxacin resistance phenotypes within the microbial community. Moreover, we revealed the quantitative relationship that metal concentrations reduced to one-third of initial levels still maintain ARG-enriching potential, providing empirical evidence for biological co-selection effects in remediated soils. This finding challenges regulatory frameworks developed primarily for acute toxicity rather than evolutionary selective pressure.

From a One Health perspective, which emphasizes the interconnectedness of human, animal, and environmental health, our results show how environmental interventions intended to protect human health may create new risks. The enrichment of multidrug-resistant bacteria in remediated soils destined for human contact represents a previously unrecognized component of environment as a source and a sink of AMR. This finding is particularly relevant for urban environments where remediated sites frequently become parks, playgrounds, and residential areas with high human exposure potential.

### Mechanisms contributing to the elevated AMR risk in the heavy-metal remediated soil

This rise in AMR-associated health risks may be attributed to two key mechanisms. First, chemical remediation reduced the concentrations of bioavailable heavy metals to levels below the inhibitory concentrations. Previous studies have shown that such sub-lethal concentrations of heavy metals can enhance bacterial resistance to multiple antibiotics [43].

In our work, a strong positive correlation was found between cadmium-resistant and ciprofloxacin-resistant bacterial activities. Our phenotypic-associated MAG analysis further revealed that 100% of cadmium resistance-associated MAGs harbored multidrug resistance genes, with some MAGs with co-resistance potential (physically linked ARG–MRG determinants on the same contigs within a distance of $\le$5 kb) that show consistent increase across nearly all samples throughout the remediation process (Table S6). These findings provide compelling evidence for metal-antibiotic co-resistance, and MRB serve as obligate reservoirs for antibiotic resistance occurrence in remediated environments. Therefore, residual low concentrations of bioavailable heavy metals in soil can continue to impose strong co-selection pressure on ARGs.

The preferential co-selection of multidrug over quinolone resistance genes by residual heavy metal reflects specific underlying mechanisms. Multidrug efflux systems can simultaneously transport diverse antibiotics and heavy metals through overlapping binding sites, providing broader adaptive advantages under multi-stress conditions [44]. Although this mechanistic framework may explain why multidrug resistance genes increased whereas quinolone-specific genes declined, residual heavy metal-mediated co-selection could still favor chromosomal mutations in quinolone target enzymes (e.g. DNA gyrase and topoisomerase IV), which would not be captured by quinolone-specific ARG profiles.

Another contributing factor to elevated AMR risk is the sustained enrichment of specific antibiotic-resistant bacteria (ARB) (Fig. 5) during the remediation process. The improved soil physicochemical conditions following remediation—such as increased availability of NH₄^+^—may have promoted microbial growth, thereby facilitating the proliferation of ARB (Table S9). Moreover, we observed a dramatic increase in the abundance of *Thermithiobacillaceae*, a taxon that was nearly undetectable initially but became dominant by the end of remediation. These populations carried extensive arsenals of ARGs and MRGs, highlighting that remediation can inadvertently select for multidrug-resistant taxa with specialized metabolic traits. This selective enrichment coincided with increased multidrug resistance gene abundance, as these sulfur-oxidizing bacteria possessed both metabolic advantages under remediation conditions and genetic arsenals for multi-stress resistance [45].

Given that low concentrations of bioavailable heavy metals can still exert strong co-selection pressure on ARGs, AMR risk assessments should not be confined to conventionally recognized heavy metal-contaminated environments. Instead, AMR surveillance should be extended to less conspicuous scenarios of low-level metal inputs, such as monitoring resistance evolution in agricultural soils subjected to metal-based pesticide pressure. Our demonstration that residual subtoxic metal concentrations maintain ARG-enriching potential also has implications for understanding resistance evolution in natural environments experiencing chronic low-level contamination.

### Study limitations and future directions

Several limitations should be acknowledged that suggest directions for future research. Although three levels of heavy-metal-contaminated soils were comparatively analyzed, all samples were derived from a single site. Thus, despite the laboratory-scale design, the findings may not fully capture field-scale complexity or site-to-site variability—including spatial heterogeneity and seasonal dynamics—highlighting the need for multi-site, long-term field validation studies. The focus on cadmium may not represent the full spectrum of metal-resistance interactions in polymetallic sites, and the 120-day timeframe may not reveal longer-term evolutionary trajectories of resistance gene persistence. Future research should focus on developing predictive models linking metal bioavailability to resistance gene dynamics, investigating resistance reversal mechanisms in metal-stressed environments, and applying emerging single-cell genomics technologies for better resolution of resistance expression patterns.

## Conclusions

This study shows that successful chemical decontamination of heavy metal-contaminated sites does not eliminate AMR risks but may enhance them through persistent co-resistance mechanisms operating at reduced metal concentrations. The universal carriage of antibiotic resistance genes by bacteria associated with Cd-resistance phenotypes in this study reveals a previously unrecognized risk in organisms that are otherwise considered environmentally beneficial.

The integration of single-cell activity measurements with genomic analysis provides a methodological foundation for improved biological risk assessment that captures resistance dynamics missed by conventional approaches. As society increasingly relies on environmental remediation to reclaim contaminated lands, understanding and managing the AMR risks associated with contamination becomes as important as addressing chemical contamination itself.

## Supplementary Material

Supplemental_Material_wrag058

## Data Availability

All data supporting the findings of this study are included in the paper and its Supplementary Materials. Raw metagenomic sequencing data are available in the NCBI Sequence Read Archive (SRA) under BioProject accession number PRJNA1332155.
