# Supplementary material for: Persistent antimicrobial resistance during soil remediation driven by residual heavy metal co-selection
Source: ISME J. 2026 Mar 18;20(1):wrag058. doi: 10.1093/ismejo/wrag058 (PMC13077299; doi:10.1093/ismejo/wrag058)
Supplement: Supplemental_Material_wrag058 [file supplemental_material_wrag058.docx]

**Supporting Information**

**Persistent antimicrobial resistance during soil remediation driven by residual heavy metal co-selection**

Rui Xue ^1, 2 #^, Yiyue Zhang ^1, 3, 4 #^, Hongzhe Li ^5^, Jian Li ^6^, Wenshun Ke ^7^, Shilin Hu ^1, 8^, Chaoran Li ^9^, Faith Ka Shun Chan ^10^, Li Cui ^1, *^

*^1^State Key Laboratory of Regional and Urban Ecology, Institute of Urban Environment, Chinese Academy of Sciences, Xiamen, Fujian 361021, China;*

*^2^College of Resources and Environment, University of Chinese Academy of Sciences, 19A Yuquan Road, Beijing 100049, China;*

*^3^State Key Laboratory of Regional and Urban Ecology, Ningbo Observation and Research Station, Institute of Urban Environment, Chinese Academy of Sciences, Xiamen, Fujian 361021, China;*

*^4^Zhejiang Key Laboratory of Pollution Control for Port-Petrochemical Industry, CAS Haixi Industrial Technology Innovation Center in Beilun, Ningbo, Zhejiang 315830, China;*

*^5^State Key Laboratory of Regional and Urban Ecology, Research Centre for Eco-Environmental Sciences, Chinese Academy of Sciences, Beijing 100085, China;*

*^6^Ministry of Education Key Laboratory of Ecology and Resource Use of the Mongolian Plateau & Inner Mongolia Key Laboratory of Grassland Ecology, School of Ecology and Environment, Inner Mongolia University, Hohhot, Inner Mongolia 010021, China;*

*^7^College of Environment and Ecology, Hunan Agricultural University, Changsha, Hunan 410128, China;*

*^8^Department of Environmental Science and Engineering, University of Science and Technology of China, Hefei, Anhui 230026, China;*

*^9^State Key Laboratory of Crop Stress Adaptation and Improvement, Henan University, Kaifeng, Henan 475004, China;*

*^10^School of Geographical Sciences, University of Nottingham Ningbo China, Ningbo, Zhejiang 315100, China;*

*^#^These authors contributed equally to this work.*

*Corresponding author. Li Cui, State Key Laboratory of Regional and Urban Ecology, Institute of Urban Environment, Chinese Academy of Sciences, 1799 Jimei road, Xiamen, Fujian 361021, China. E-mail addresses: [lcui@iue.ac.cn](mailto:lcui@iue.ac.cn).

**This file includes:**

Supplementary Figures 1 to 11

Supplementary Tables 1 to 9

**Supplementary Figures**


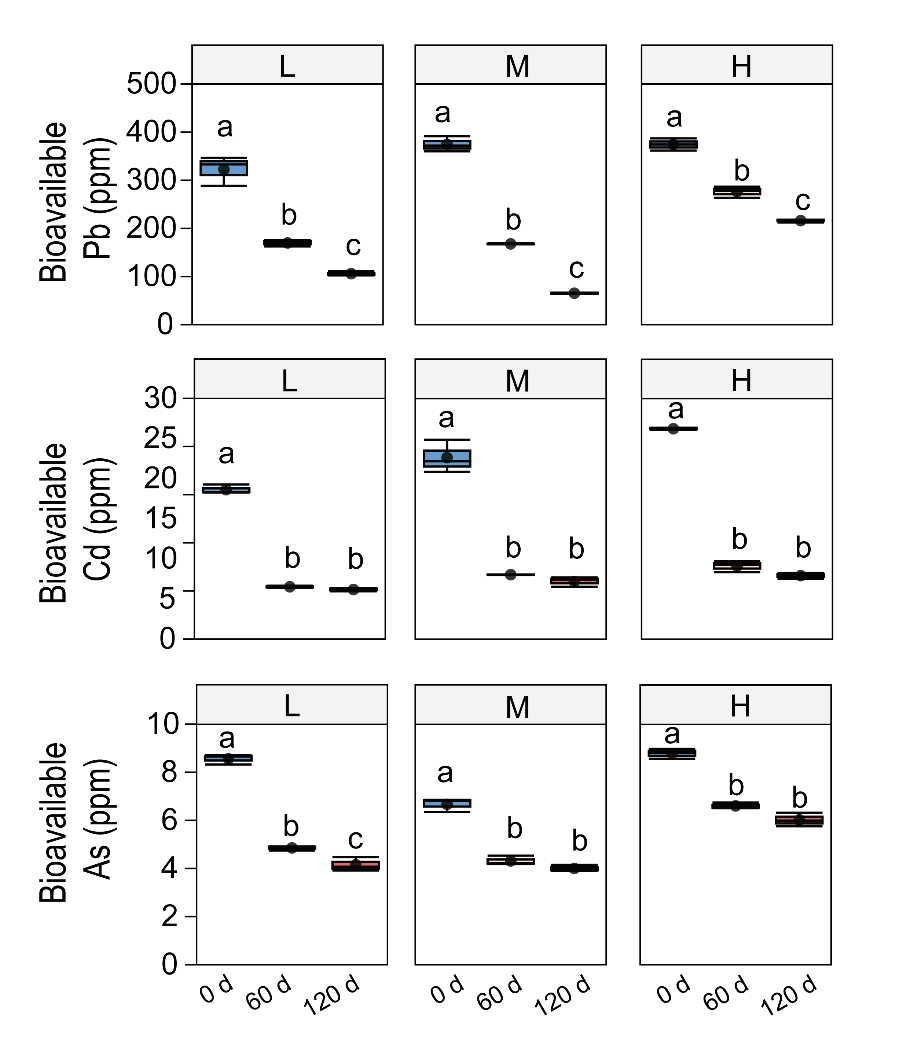


**Fig. S1.** Impact of remediation on bioavailable heavy metals. Concentrations of bioavailable Pb, Cd, and As in slightly (L), moderately (M), and heavily (H) contaminated soils throughout the remediation process. Different letters indicate significant differences based on one-way ANOVA followed by Tukey's HSD test (*P* < 0.05).


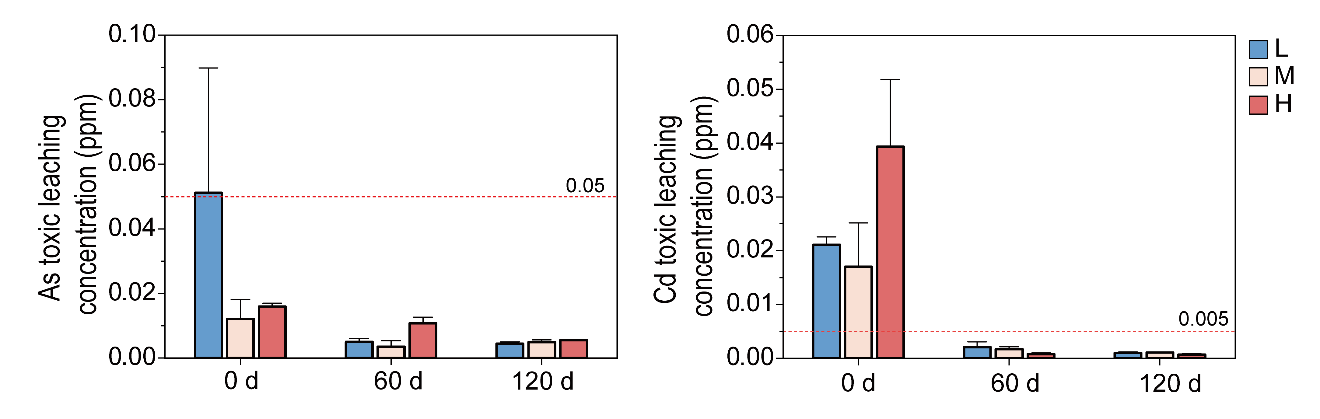


**Fig. S2.** Impact of remediation on heavy metal leaching toxicity concentrations. Data are presented as mean ± standard deviation (n = 3). The red dashed line indicates the threshold values for Class III surface water quality limits according to China's GB3838-2002 standard. Leaching toxicity of Pb was below the detection limit in all samples. Heavy metal leaching toxicity primarily reflects the potential risk of heavy metals being mobilized through rainfall leaching or water-mediated transport in soils.


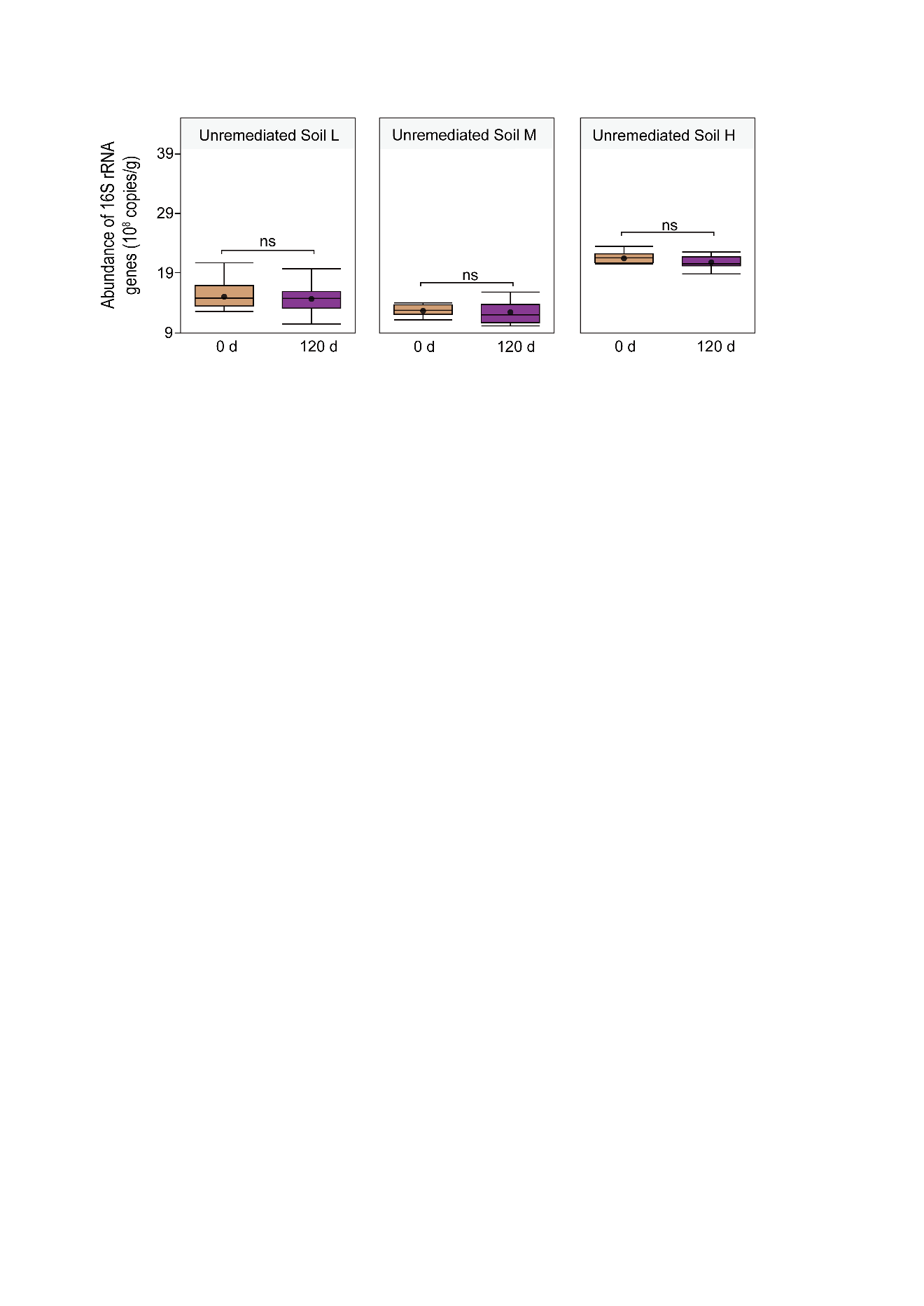


**Fig. S3.** Absolute abundance of 16S rRNA genes in the unremediated soil (0 d) and the non-remediated temporal control (120 d). NS (ns) denote no significant differences between 0 d and 120 d (*P* > 0.05, two-tailed Student's t-test).


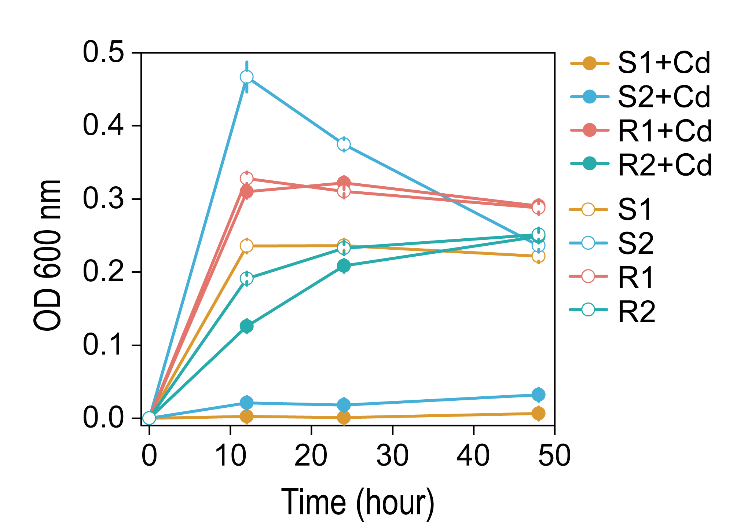


**Fig. S4.** OD₆₀₀ values of four bacterial strains cultured in R2A media with 50% D₂O, with or without cadmium (R2A-Cd and R2A-NoCd), measured at 0, 12, 24, and 48 h. S1 and S2 represent cadmium-sensitive strains *Escherichia coli* and *Bacillus cereus*, respectively, while R1 and R2 correspond to cadmium-resistant strains *Cupriavidus nantongensis* and *Pseudomonas ceruminis*.


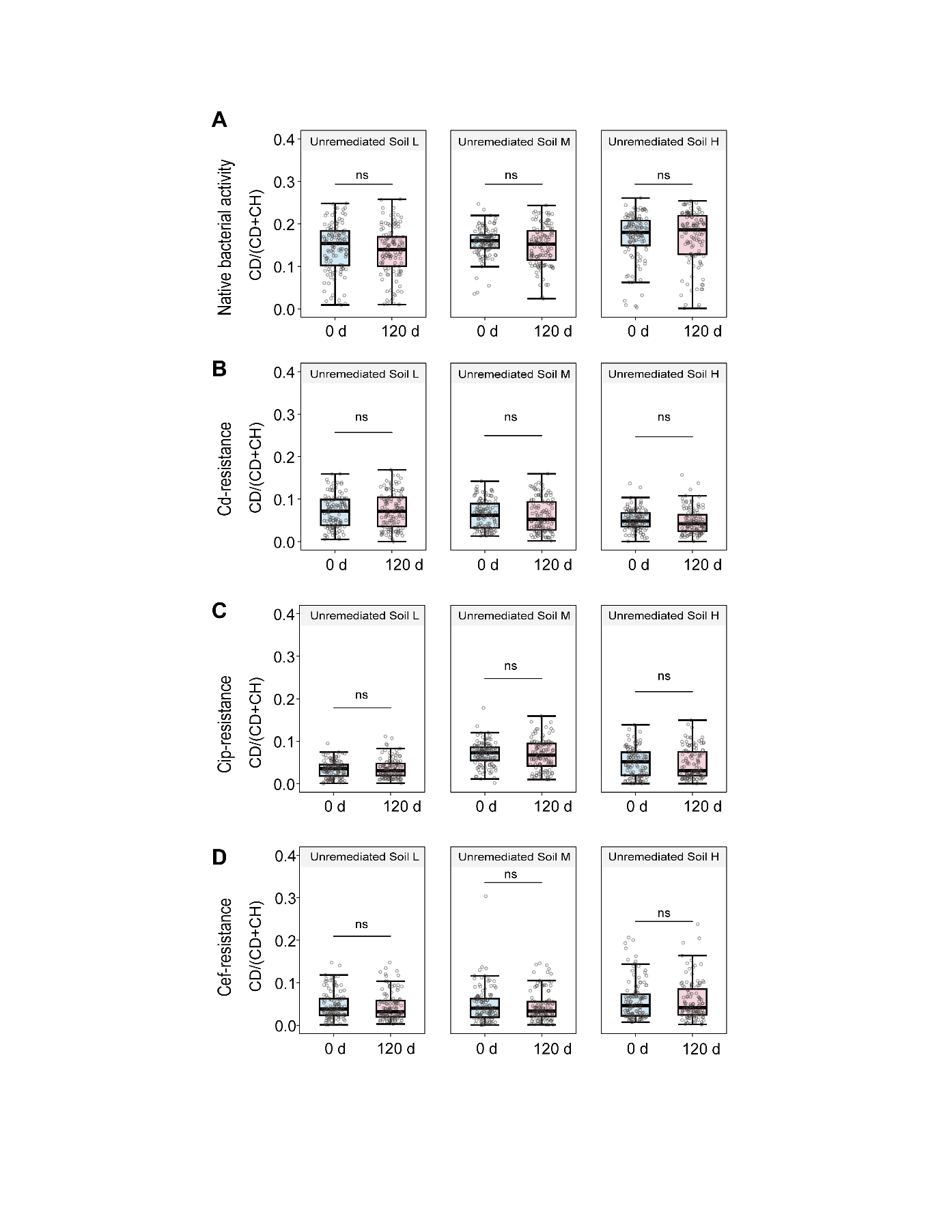


**Fig. S5.** Phenotypic activity of distinct bacterial populations in the unremediated soil (0 d) and the non-remediated temporal control (120 d). **(A**–**D)** Metabolic activity profiles of four bacterial phenotypes: (**A**) native active bacteria, (B) cadmium-resistant bacteria, (**C**) ciprofloxacin-resistant bacteria, and (**D**) cefotaxime-resistant bacteria. Activity quantified using C–D ratios from single-cell Raman spectroscopy following D₂O labeling. NS (ns) denote no significant differences between 0 d and 120 d (*P* > 0.05, two-tailed Student's t-test). The absence of significant differences confirms that resistance enrichment observed in remediated soils results from remediation processes rather than laboratory artifacts.


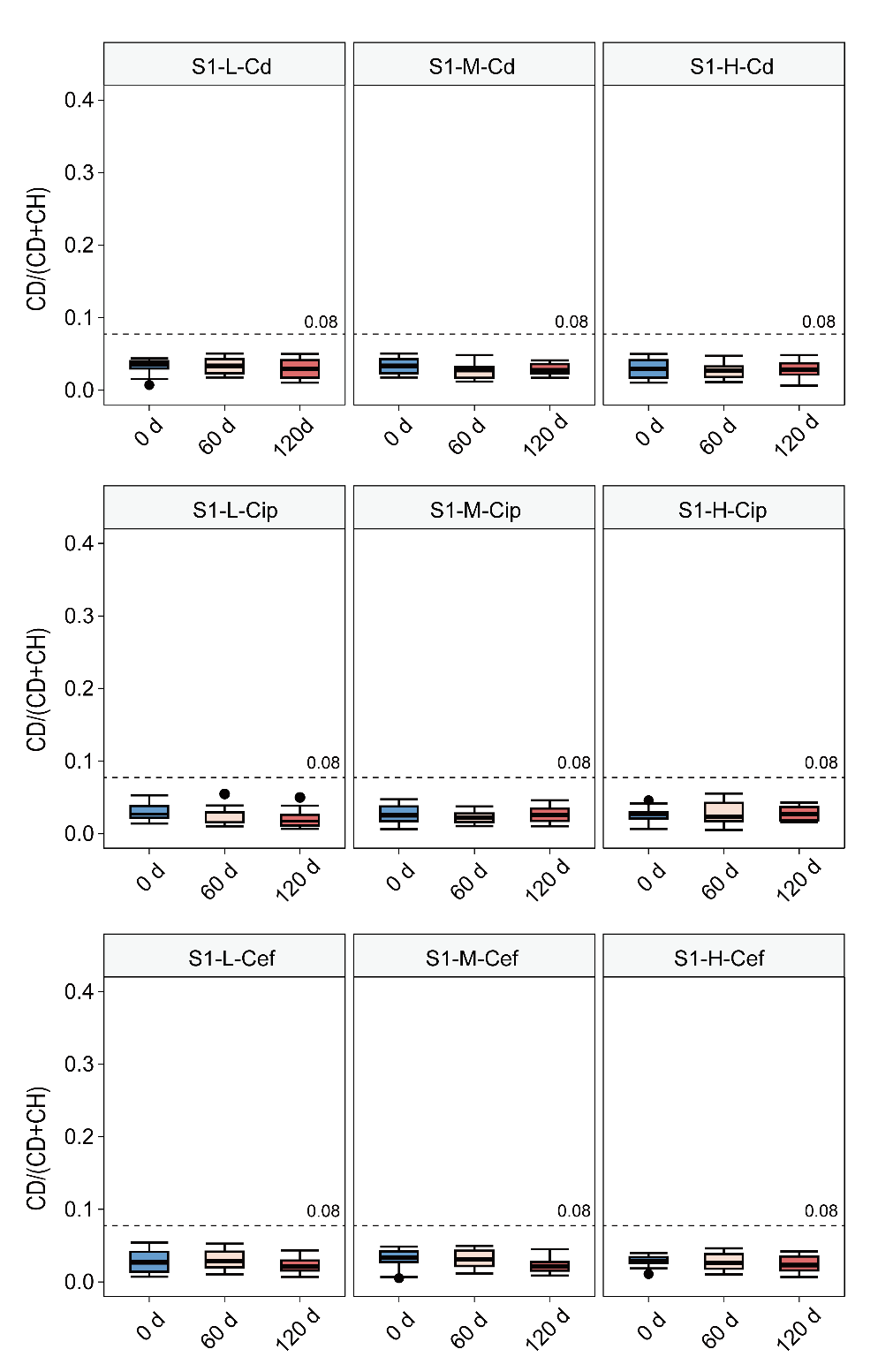


**Fig. S6.** Raman-based phenotypic profiling of the sensitive strain S1 (*Escherichia coli*) in sterilized soil samples, assessing resistance to cadmium (Cd), ciprofloxacin (Cip), and ceftazidime (Cef). To exclude false-positive signals resulting from potential adsorption of heavy metals or antibiotics by the remediation materials–leading to apparent metabolic activity in sensitive strains–S1, which is sensitive to both cadmium and antibiotics, was added to all sterilized soil samples under the same labeling conditions. The gray dashed line represents the metabolic activity threshold.


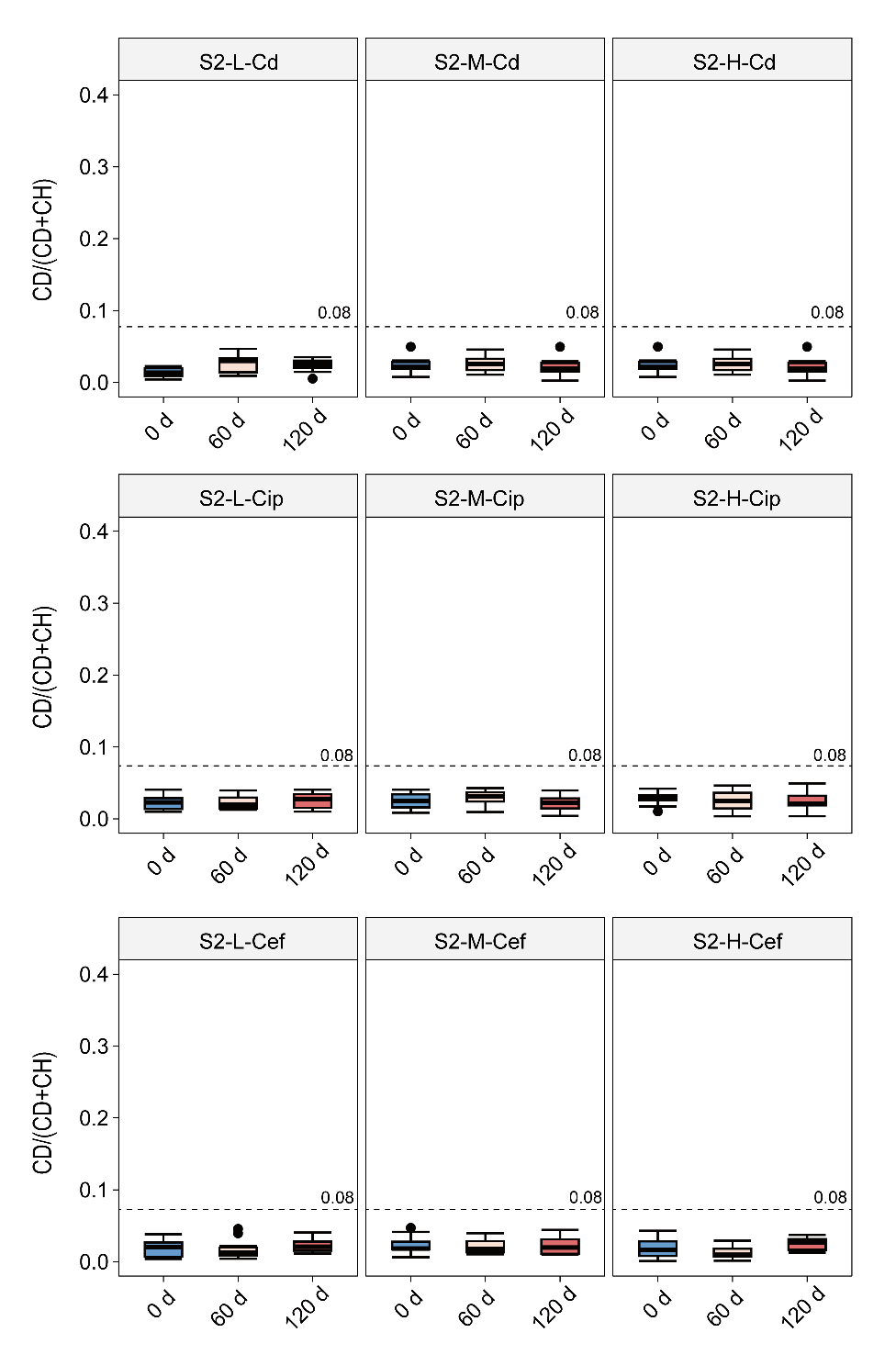


**Fig. S7.** Raman-based phenotypic profiling of the sensitive strain S2 (*Bacillus cereus*) in sterilized soil samples, assessing resistance to cadmium (Cd), ciprofloxacin (Cip), and ceftazidime (Cef). To exclude false-positive signals resulting from potential adsorption of heavy metals or antibiotics by the remediation materials—leading to apparent metabolic activity in sensitive strains–S2, which is sensitive to both cadmium and antibiotics, was added to all sterilized soil samples under the same labeling conditions. The gray dashed line represents the metabolic activity threshold.


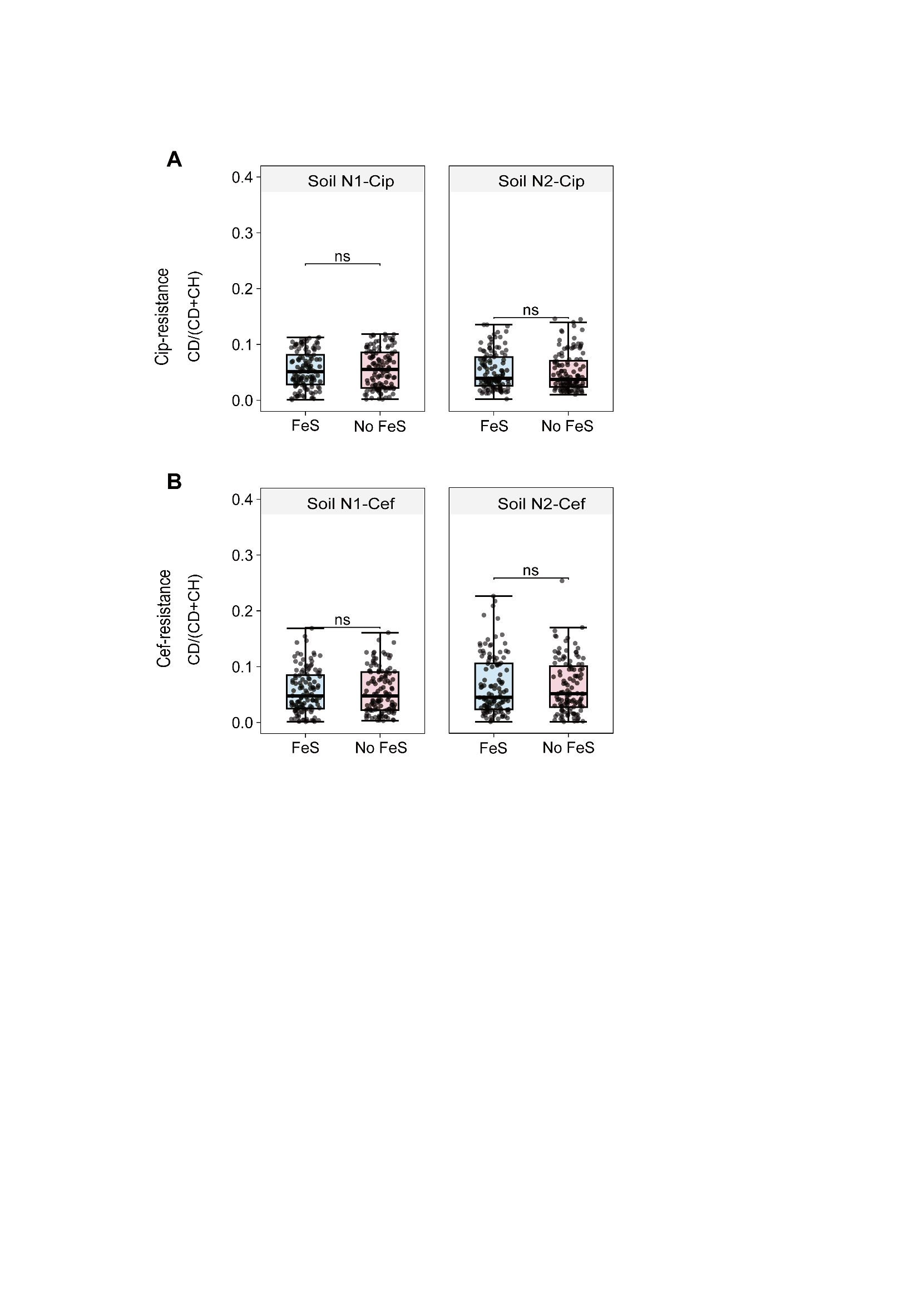


**Fig. S8. Phenotypic activity of antibiotic-resistant bacteria in uncontaminated soils (N1, N2) with and without FeS treatment over 120 days.** (A) ciprofloxacin-resistant bacteria; (B) cefotaxime-resistant bacteria. Bacterial activity was quantified using C–D ratios obtained from single-cell Raman spectroscopy following D₂O labeling. NS (ns) indicates no significant difference between the FeS and No FeS groups (*P* > 0.05, two-tailed Student's t-test).


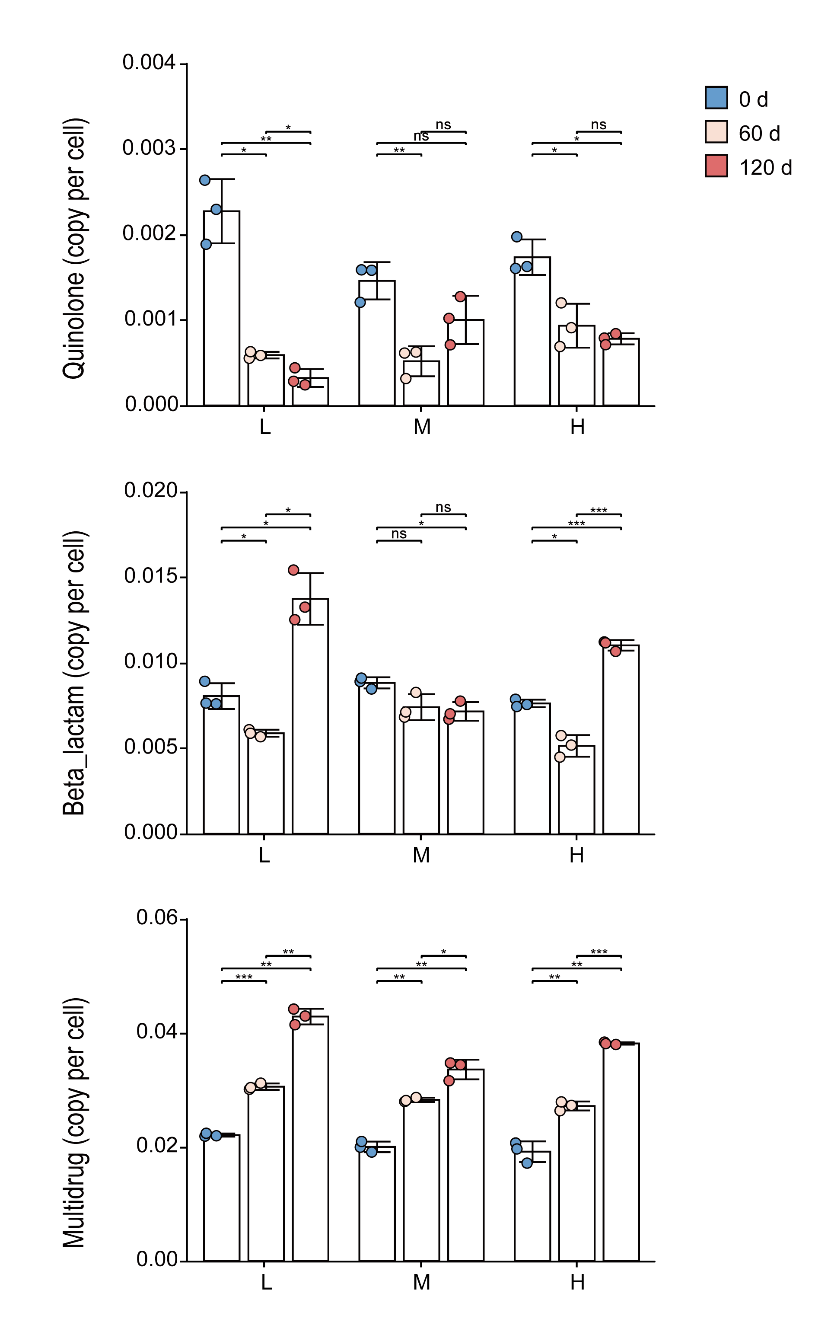


**Fig. S9.** Relative abundance of phenotype-associated antibiotic resistance genes (ARGs) across different soil treatments. Bar plots show the normalized abundance of ARGs per cell associated with quinolone, β-lactam, and multidrug resistance in slightly (L), moderately (M), and heavily (H) contaminated soils. Asterisks indicate significant differences among time points within each soil type (one-way ANOVA followed by Tukey's HSD test: **P* < 0.05, ***P* < 0.01, **P* < 0.001; ns, not significant).


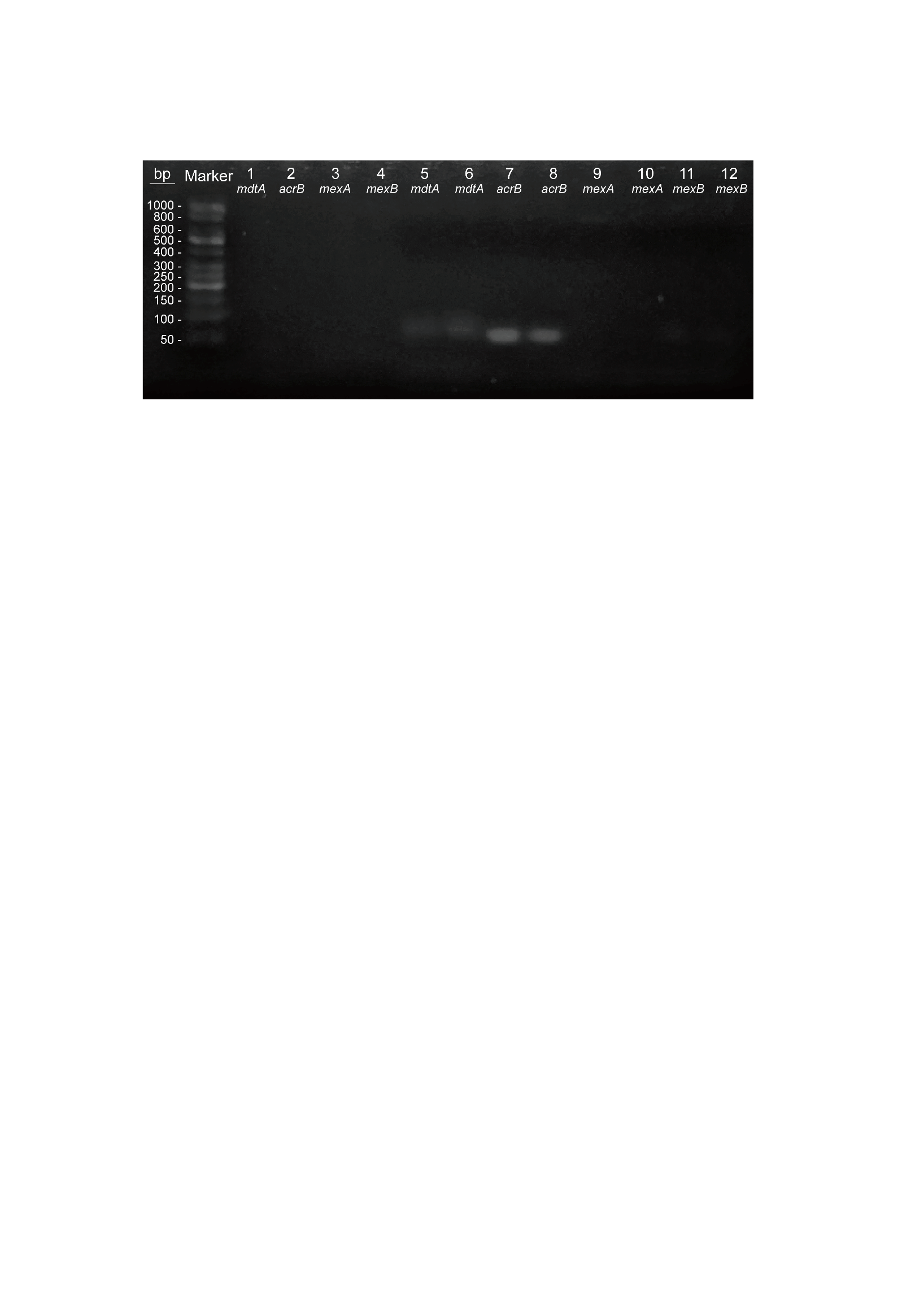


**Fig. S10.** Identification of multidrug resistance genes in cadmium resistant bacterial isolates by PCR. Lanes: 1–4, no-template DNA controls; 5, 7, 9, and 11, *Cupriavidus nantongensis*; 6, 8, 10, and 12, *Microbacterium marinilacus*. Expected amplicon sizes were 82 bp for *mdtA*, 65 bp for *acrB*, 79 bp for *mexA*, and 102 bp for *mexB*. Both isolates carried the multidrug resistance genes *mdtA* and *acrB*.


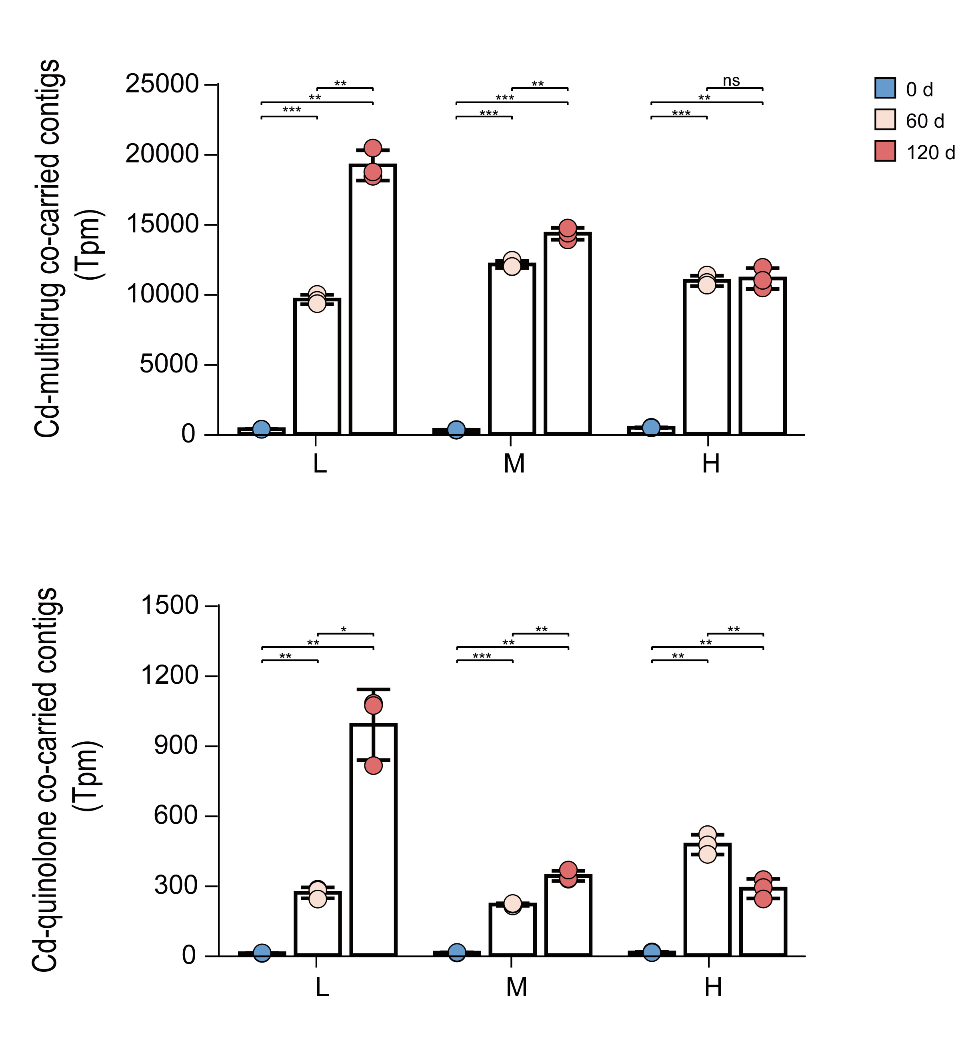


**Fig. S11.** Abundance of ARG–MRG co-carrying contigs (MAG-based) across different soil samples. TPM (transcripts per million) denotes the abundance of ARG–MRG co-carried contigs. Bars represent the relative abundance of contigs harboring both antibiotic resistance genes (ARGs) and metal resistance genes (MRGs). Asterisks indicate significant differences among time points within each soil type (one-way ANOVA followed by Tukey's HSD test: **P* < 0.05, ***P* < 0.01, **P* < 0.001; ns, not significant). Cd: cadmium resistance genes; Multidrug: multidrug resistance genes; Quinolone: quinolone resistance genes.

**Supplementary Tables**

**Table S1. Total concentrations of heavy metals in the soil of L, M, H, N1 and N2.**

| Content | L | M | H | N1 | N2 |
| --- | --- | --- | --- | --- | --- |
| Cd (mg kg^-1^) | 165.0$\pm12.40c$ | 230.0$\pm7.363$b | 433.6$\pm13.28a$ | $0.05\pm0.0092e$ | $0.22\pm0.016d$ |
| Pb (mg kg^-1^) | 1949$\pm80.87$a | 2330$\pm129.6$b | 2856$\pm102.3$c | $2.2\pm0.41e$ | $6.4\pm0.69d$ |
| As (mg kg^-1^) | 500.5$\pm70.24$a | 798.7$\pm44.82$b | 967.4$\pm41.19$c | $0.15\pm0.036e$ | $0.52\pm0.029d$ |

Different letters indicate significant differences (*P* < 0.05, one-way ANOVA followed by Tukey's HSD test).

**Table S2. Genomic co-localization of antibiotic resistance genes (ARGs) and cadmium resistance genes (CdRGs) on contigs within metagenome-assembled genomes (MAGs) from phenotypic resistance communities.**

| MAG | contig in MAG | length | ARG | ARG_start | ARG_end | ARG_+/- | CdRG | CdRG_start | CdRG_end | CdRG_+/- |
| --- | --- | --- | --- | --- | --- | --- | --- | --- | --- | --- |
| S12C60 | gnl\|S12C60\|S12C60_11 | 453221 | *mexW* | 436120 | 439248 | - | *czrB* | 439258 | 440427 | - |
| S1C60 | gnl\|S1C60\|S1C60_15 | 142518 | *mexW* | 125417 | 128545 | - | *czrB* | 128555 | 129724 | - |
| S12C157 | gnl\|S12C157\|S12C157_30 | 10149 | *mdtC* | 5677 | 8856 | + | *czcP* | 3092 | 5632 | + |
| S13C157 | gnl\|S13C157\|S13C157_42 | 126287 | *mdtC* | 65432 | 68611 | - | *czcP* | 68656 | 71196 | - |
| S14C157 | gnl\|S14C157\|S14C157_57 | 270889 | *mdtC* | 210034 | 213213 | - | *czcP* | 213258 | 215798 | - |
| S15C157 | gnl\|S15C157\|S15C157_43 | 270889 | *mdtC* | 210034 | 213213 | - | *czcP* | 213258 | 215798 | - |
| S16C157 | gnl\|S16C157\|S16C157_144 | 30638 | *mdtC* | 9 | 3188 | - | *czcP* | 3233 | 5773 | - |
| S17C157 | gnl\|S17C157\|S17C157_43 | 93532 | *mdtC* | 62951 | 66130 | - | *czcP* | 66175 | 68715 | - |
| S18C157 | gnl\|S18C157\|S18C157_91 | 31042 | *mdtC* | 27471 | 30650 | + | *czcP* | 24886 | 27426 | + |
| S1C157 | gnl\|S1C157\|S1C157_59 | 10148 | *mdtC* | 1294 | 4473 | - | *czcP* | 4518 | 7058 | - |
| S16C363 | gnl\|S16C363\|S16C363_10 | 312854 | *multidrug_ABC_transporter* | 297637 | 298467 | - | *irlR* | 298885 | 299880 | + |
| S17C363 | gnl\|S17C363\|S17C363_17 | 312854 | *multidrug_ABC_transporter* | 297637 | 298467 | - | *irlR* | 298885 | 299880 | + |
| S18C363 | gnl\|S18C363\|S18C363_54 | 137735 | *multidrug_ABC_transporter* | 14388 | 15218 | + | *irlR* | 12975 | 13970 | - |
| S19C363 | gnl\|S19C363\|S19C363_53 | 312854 | *multidrug_ABC_transporter* | 297637 | 298467 | - | *irlR* | 298885 | 299880 | + |
| S20C363 | gnl\|S20C363\|S20C363_25 | 312854 | *multidrug_ABC_transporter* | 297637 | 298467 | - | *irlR* | 298885 | 299880 | + |
| S12C157 | gnl\|S12C157\|S12C157_51 | 33758 | *multidrug_transporter* | 32417 | 33679 | + | *nmtR* | 31134 | 31472 | - |
| S13C157 | gnl\|S13C157\|S13C157_52 | 33758 | *multidrug_transporter* | 32417 | 33679 | + | *nmtR* | 31134 | 31472 | - |
| S14C157 | gnl\|S14C157\|S14C157_53 | 33758 | *multidrug_transporter* | 32417 | 33679 | + | *nmtR* | 31134 | 31472 | - |
| S15C157 | gnl\|S15C157\|S15C157_44 | 33758 | *multidrug_transporter* | 32417 | 33679 | + | *nmtR* | 31134 | 31472 | - |
| S17C157 | gnl\|S17C157\|S17C157_76 | 33758 | *multidrug_transporter* | 32417 | 33679 | + | *nmtR* | 31134 | 31472 | - |
| S18C157 | gnl\|S18C157\|S18C157_283 | 12805 | *multidrug_transporter* | 11464 | 12726 | + | *nmtR* | 10181 | 10519 | - |
| S1C157 | gnl\|S1C157\|S1C157_94 | 33758 | *multidrug_transporter* | 32417 | 33679 | + | *nmtR* | 31134 | 31472 | - |
| S12C157 | gnl\|S12C157\|S12C157_30 | 10149 | *mexF* | 7 | 1992 | + | *czcP* | 3092 | 5632 | + |
| S13C157 | gnl\|S13C157\|S13C157_42 | 126287 | *mexF* | 72296 | 75538 | - | *czcP* | 68656 | 71196 | - |
| S14C157 | gnl\|S14C157\|S14C157_57 | 270889 | *mexF* | 216898 | 220140 | - | *czcP* | 213258 | 215798 | - |
| S15C157 | gnl\|S15C157\|S15C157_43 | 270889 | *mexF* | 216898 | 220140 | - | *czcP* | 213258 | 215798 | - |
| S16C157 | gnl\|S16C157\|S16C157_144 | 30638 | *mexF* | 6873 | 10115 | - | *czcP* | 3233 | 5773 | - |
| S17C157 | gnl\|S17C157\|S17C157_43 | 93532 | *mexF* | 69815 | 73057 | - | *czcP* | 66175 | 68715 | - |
| S18C157 | gnl\|S18C157\|S18C157_91 | 31042 | *mexF* | 20544 | 23786 | + | *czcP* | 24886 | 27426 | + |
| S1C157 | gnl\|S1C157\|S1C157_59 | 10148 | *mexF* | 8158 | 10143 | - | *czcP* | 4518 | 7058 | - |
| S13C157 | gnl\|S13C157\|S13C157_42 | 126287 | *mexF* | 72296 | 75538 | - | *czcC* | 76821 | 78128 | - |
| S14C157 | gnl\|S14C157\|S14C157_57 | 270889 | *mexF* | 216898 | 220140 | - | *czcC* | 221423 | 222730 | - |
| S15C157 | gnl\|S15C157\|S15C157_43 | 270889 | *mexF* | 216898 | 220140 | - | *czcC* | 221423 | 222730 | - |
| S16C157 | gnl\|S16C157\|S16C157_144 | 30638 | *mexF* | 6873 | 10115 | - | *czcC* | 11398 | 12705 | - |
| S17C157 | gnl\|S17C157\|S17C157_43 | 93532 | *mexF* | 69815 | 73057 | - | *czcC* | 74340 | 75647 | - |
| S18C157 | gnl\|S18C157\|S18C157_91 | 31042 | *mexF* | 20544 | 23786 | + | *czcC* | 17954 | 19261 | + |
| S12C60 | gnl\|S12C60\|S12C60_11 | 453221 | *mexW* | 436120 | 439248 | - | *czcD* | 433810 | 434778 | - |
| S1C60 | gnl\|S1C60\|S1C60_15 | 142518 | *mexW* | 125417 | 128545 | - | *czcD* | 123107 | 124075 | - |
| S16C363 | gnl\|S16C363\|S16C363_28 | 97314 | *mdtB* | 25879 | 29016 | - | *actR* | 23534 | 24142 | - |
| S17C363 | gnl\|S17C363\|S17C363_72 | 97315 | *mdtB* | 25880 | 29017 | - | *actR* | 23535 | 24143 | - |
| S18C363 | gnl\|S18C363\|S18C363_29 | 97315 | *mdtB* | 25880 | 29017 | - | *actR* | 23535 | 24143 | - |
| S19C363 | gnl\|S19C363\|S19C363_18 | 97315 | *mdtB* | 25880 | 29017 | - | *actR* | 23535 | 24143 | - |
| S20C363 | gnl\|S20C363\|S20C363_1 | 97314 | *mdtB* | 25879 | 29016 | - | *actR* | 23534 | 24142 | - |
| S12C60 | gnl\|S12C60\|S12C60_7 | 184826 | *multidrug_ABC_transporter* | 171145 | 172983 | - | *czcA* | 174727 | 177822 | - |
| S1C60 | gnl\|S1C60\|S1C60_16 | 184826 | *multidrug_ABC_transporter* | 171145 | 172983 | - | *czcA* | 174727 | 177822 | - |
| S12C157 | gnl\|S12C157\|S12C157_32 | 163781 | *emrB* | 122057 | 123352 | - | *fpvA* | 125171 | 127438 | - |
| S13C157 | gnl\|S13C157\|S13C157_48 | 101975 | *emrB* | 60251 | 61546 | - | *fpvA* | 63365 | 65632 | - |
| S14C157 | gnl\|S14C157\|S14C157_39 | 163781 | *emrB* | 122057 | 123352 | - | *fpvA* | 125171 | 127438 | - |
| S15C157 | gnl\|S15C157\|S15C157_9 | 70146 | *emrB* | 40430 | 41725 | + | *fpvA* | 36344 | 38611 | + |
| S16C157 | gnl\|S16C157\|S16C157_133 | 5923 | *emrB* | 4485 | 5780 | + | *fpvA* | 399 | 2666 | + |
| S17C157 | gnl\|S17C157\|S17C157_192 | 13410 | *emrB* | 9925 | 11220 | + | *fpvA* | 5839 | 8106 | + |
| S18C157 | gnl\|S18C157\|S18C157_1 | 46433 | *emrB* | 23612 | 24907 | - | *fpvA* | 26726 | 28993 | - |
| S1C157 | gnl\|S1C157\|S1C157_75 | 101970 | *emrB* | 60246 | 61541 | - | *fpvA* | 63360 | 65627 | - |
| S12C177 | gnl\|S12C177\|S12C177_34 | 80601 | *multidrug_transporter* | 61424 | 65776 | + | *cadD* | 69650 | 70243 | + |
| S13C177 | gnl\|S13C177\|S13C177_67 | 80601 | *multidrug_transporter* | 61424 | 65776 | + | *cadD* | 69650 | 70243 | + |
| S14C177 | gnl\|S14C177\|S14C177_53 | 80601 | *multidrug_transporter* | 61424 | 65776 | + | *cadD* | 69650 | 70243 | + |
| S15C177 | gnl\|S15C177\|S15C177_5 | 80433 | *multidrug_transporter* | 61424 | 65776 | + | *cadD* | 69650 | 70243 | + |
| S1C177 | gnl\|S1C177\|S1C177_18 | 80650 | *multidrug_transporter* | 61424 | 65776 | + | *cadD* | 69650 | 70243 | + |
| S13C157 | gnl\|S13C157\|S13C157_42 | 126287 | *cmeB* | 82342 | 85503 | + | *czcC* | 76821 | 78128 | - |
| S14C157 | gnl\|S14C157\|S14C157_57 | 270889 | *cmeB* | 226944 | 230105 | + | *czcC* | 221423 | 222730 | - |
| S15C157 | gnl\|S15C157\|S15C157_43 | 270889 | *cmeB* | 226944 | 230105 | + | *czcC* | 221423 | 222730 | - |
| S16C157 | gnl\|S16C157\|S16C157_144 | 30638 | *cmeB* | 16919 | 20080 | + | *czcC* | 11398 | 12705 | - |
| S17C157 | gnl\|S17C157\|S17C157_43 | 93532 | *cmeB* | 79861 | 83022 | + | *czcC* | 74340 | 75647 | - |
| S18C157 | gnl\|S18C157\|S18C157_91 | 31042 | *cmeB* | 10579 | 13740 | - | *czcC* | 17954 | 19261 | + |
| S12C209 | gnl\|S12C209\|S12C209_41 | 90813 | *mexF* | 11179 | 14343 | + | *irlS* | 18585 | 19973 | + |
| S13C209 | gnl\|S13C209\|S13C209_33 | 254550 | *mexF* | 240208 | 243372 | - | *irlS* | 234578 | 235966 | - |
| S14C209 | gnl\|S14C209\|S14C209_98 | 35958 | *mexF* | 11179 | 14343 | + | *irlS* | 18585 | 19973 | + |
| S15C209 | gnl\|S15C209\|S15C209_111 | 254550 | *mexF* | 240208 | 243372 | - | *irlS* | 234578 | 235966 | - |
| S1C209 | gnl\|S1C209\|S1C209_2 | 254550 | *mexF* | 240208 | 243372 | - | *irlS* | 234578 | 235966 | - |
| S10C696 | gnl\|S10C696\|S10C696_17 | 94361 | *multidrug_transporter* | 86735 | 89833 | + | *cadR* | 81511 | 81924 | - |
| S12C696 | gnl\|S12C696\|S12C696_7 | 93980 | *multidrug_transporter* | 86735 | 89833 | + | *cadR* | 81511 | 81924 | - |
| S13C696 | gnl\|S13C696\|S13C696_20 | 93980 | *multidrug_transporter* | 86735 | 89833 | + | *cadR* | 81511 | 81924 | - |
| S14C696 | gnl\|S14C696\|S14C696_9 | 93980 | *multidrug_transporter* | 86735 | 89833 | + | *cadR* | 81511 | 81924 | - |
| S1C696 | gnl\|S1C696\|S1C696_1 | 93980 | *multidrug_transporter* | 86735 | 89833 | + | *cadR* | 81511 | 81924 | - |

**Table S3. The abundance of ARGs detected in this study.**

| ARG Type | L 0 day | L 60 day | L 120 day | M 0 day | M 60 day | M 120 day | H 0 day | H 60 day | H 120 day |
| --- | --- | --- | --- | --- | --- | --- | --- | --- | --- |
| \| multidrug \| \| --- \| \| bacitracin \| \| polymyxin \| \| rifamycin \| \| novobiocin \| \| mls \| \| vancomycin \| \| mupirocin \| \| beta_lactam \| \| tetracycline \| \| tetracenomycin_C \| \| aminoglycoside \| \| chloramphenicol \| \| quinolone \| \| sulfonamide \| \| fosfomycin \| \| pleuromutilin_tiamulin \| \| trimethoprim \| \| florfenicol \| \| defensin \| \| puromycin \| \| other_peptide_antibiotics \| \| streptothricin \| \| bleomycin \| \| antibacterial_fatty_acid \| | \| 0.02209 \| \| --- \| \| 0.021848 \| \| 0.008058 \| \| 0.015756 \| \| 0.033141 \| \| 0.042781 \| \| 0.0166 \| \| 0.004009 \| \| 0.008056 \| \| 0.009613 \| \| 0.018139 \| \| 0.003838 \| \| 0.000558 \| \| 0.002273 \| \| 0.000998 \| \| 0.000142 \| \| 0.000648 \| \| 0.000105 \| \| 5.15E-05 \| \| 1.55E-05 \| \| 5.57E-05 \| \| 7.91E-05 \| \| 4.23E-05 \| \| 0 \| \| 1.58E-05 \| | \| 0.030584 \| \| --- \| \| 0.019346 \| \| 0.00959 \| \| 0.009907 \| \| 0.00665 \| \| 0.00235 \| \| 0.003097 \| \| 0.014384 \| \| 0.005883 \| \| 0.002806 \| \| 9.08E-05 \| \| 0.001409 \| \| 0.001568 \| \| 0.000589 \| \| 0.000197 \| \| 0.001326 \| \| 0.000542 \| \| 0.000474 \| \| 0.000223 \| \| 3.70E-06 \| \| 0 \| \| 0 \| \| 0 \| \| 3.13E-05 \| \| 8.80E-06 \| | \| 0.042881 \| \| --- \| \| 0.034031 \| \| 0.018547 \| \| 0.009707 \| \| 0.003106 \| \| 0.001697 \| \| 0.001686 \| \| 0.006629 \| \| 0.013739 \| \| 0.002677 \| \| 2.92E-05 \| \| 0.00383 \| \| 0.002126 \| \| 0.000322 \| \| 0.000914 \| \| 0.000415 \| \| 0.000133 \| \| 0.000424 \| \| 0.000223 \| \| 0 \| \| 3.57E-05 \| \| 0 \| \| 9.25E-05 \| \| 0 \| \| 1.06E-05 \| | \| 0.020009 \| \| --- \| \| 0.017901 \| \| 0.015207 \| \| 0.01475 \| \| 0.019195 \| \| 0.015885 \| \| 0.032556 \| \| 0.008915 \| \| 0.00883 \| \| 0.01369 \| \| 0.00651 \| \| 0.002983 \| \| 0.000366 \| \| 0.001459 \| \| 0.00091 \| \| 0.000368 \| \| 0.000364 \| \| 0.000307 \| \| 2.19E-05 \| \| 4.73E-06 \| \| 4.23E-05 \| \| 0.000108 \| \| 4.57E-05 \| \| 2.69E-05 \| \| 2.16E-05 \| | \| 0.028273 \| \| --- \| \| 0.032537 \| \| 0.015371 \| \| 0.014739 \| \| 0.005701 \| \| 0.003502 \| \| 0.004568 \| \| 0.012737 \| \| 0.007408 \| \| 0.004516 \| \| 0.00017 \| \| 0.004328 \| \| 0.001146 \| \| 0.000518 \| \| 0.000711 \| \| 0.000726 \| \| 0.000735 \| \| 0.00055 \| \| 0.000525 \| \| 0.000264 \| \| 6.67E-06 \| \| 0 \| \| 0 \| \| 7.60E-05 \| \| 9.60E-06 \| | \| 0.033595 \| \| --- \| \| 0.03674 \| \| 0.016655 \| \| 0.010651 \| \| 0.003922 \| \| 0.003207 \| \| 0.003545 \| \| 0.008516 \| \| 0.007161 \| \| 0.003828 \| \| 0.000139 \| \| 0.006631 \| \| 0.002092 \| \| 0.001001 \| \| 0.001249 \| \| 0.00053 \| \| 0.000465 \| \| 0.000531 \| \| 0.000497 \| \| 0.000237 \| \| 0 \| \| 0 \| \| 0 \| \| 2.66E-05 \| \| 1.70E-05 \| | \| 0.019173 \| \| --- \| \| 0.016566 \| \| 0.008525 \| \| 0.01439 \| \| 0.02368 \| \| 0.024041 \| \| 0.018157 \| \| 0.004575 \| \| 0.007627 \| \| 0.011053 \| \| 0.010533 \| \| 0.003866 \| \| 0.000443 \| \| 0.001735 \| \| 0.001021 \| \| 0.000136 \| \| 0.000422 \| \| 0.000157 \| \| 3.08E-05 \| \| 2.80E-05 \| \| 9.34E-05 \| \| 7.38E-05 \| \| 0 \| \| 0 \| \| 1.44E-05 \| | \| 0.027191 \| \| --- \| \| 0.02203 \| \| 0.012455 \| \| 0.018265 \| \| 0.006092 \| \| 0.003584 \| \| 0.004808 \| \| 0.012877 \| \| 0.005142 \| \| 0.00379 \| \| 6.99E-05 \| \| 0.002211 \| \| 0.000652 \| \| 0.000934 \| \| 0.000338 \| \| 0.00152 \| \| 0.000797 \| \| 1.92E-05 \| \| 8.13E-06 \| \| 4.69E-05 \| \| 6.11E-05 \| \| 0 \| \| 0 \| \| 0 \| \| 9.40E-06 \| | \| 0.038175 \| \| --- \| \| 0.026439 \| \| 0.019608 \| \| 0.015489 \| \| 0.004302 \| \| 0.002335 \| \| 0.00248 \| \| 0.009571 \| \| 0.011023 \| \| 0.002821 \| \| 5.57E-05 \| \| 0.004668 \| \| 0.001689 \| \| 0.00078 \| \| 0.00018 \| \| 0.000803 \| \| 0.000299 \| \| 0.000396 \| \| 0.00024 \| \| 7.97E-05 \| \| 0 \| \| 0 \| \| 0 \| \| 0 \| \| 1.42E-05 \| |

**Table S4. The abundance of Cefotaxime -specific resistance genes detected in this study.**

| Cefotaxime resistance gene | L 0 day | L 60 day | L 120 day | M 0 day | M 60 day | M 120 day | H 0 day | H 60 day | H 120 day |
| --- | --- | --- | --- | --- | --- | --- | --- | --- | --- |
| *bla__SHV-41* | 0 | 0 | 0 | 1.39333E-05 | 0 | 0 | 0 | 0 | 0 |
| *bla__TEM-1* | 0 | 0 | 0 |  | 1.49333E-05 | 0 | 0 | 0 | 0 |
| *bla__TEM-117* | 0 | 0 | 0 |  | 0.0000298 | 0 | 0 | 0 | 0 |
| *bla__TEM-205* | 0 | 0 | 0 |  | 0.0000134 | 0 | 0 | 0 | 0 |
| *bla__CTX-M-3* | 0 | 0 | 0 | 0.0000126 | 0 | 0 | 0 | 0 | 0 |

**Table S5. Risk index of ARGs detected in this study.**

| ARG Class | ARO Term | Human accessibility | Mobility | Human pathogenicity | Clinical availability | risk |
| --- | --- | --- | --- | --- | --- | --- |
| aminoglycoside antibiotic | AAC(2')-Ic | 8.88E-05 | 1 | 0.934641 | 56.10526 | 0.004658 |
| aminoglycoside antibiotic | AAC(3)-Ib | 0.023556 | 25 | 0.909091 | 56.10526 | 30.03658 |
| aminoglycoside antibiotic | AAC(3)-IId | 0.648058 | 162 | 0.869482 | 56.10526 | 5121.453 |
| aminoglycoside antibiotic | AAC(3)-IIe | 0.118139 | 81 | 0.924528 | 56.10526 | 496.3671 |
| aminoglycoside antibiotic | AAC(3)-IV | 0.00323 | 68 | 0.778157 | 56.10526 | 9.589868 |
| aminoglycoside antibiotic | AAC(6')-Ib | 0.002478 | 8 | 1 | 56.10526 | 1.112329 |
| aminoglycoside antibiotic | AAC(6')-Ib' | 0.002334 | 8 | 0.166667 | 56.10526 | 0.174594 |
| aminoglycoside antibiotic | AAC(6')-Ib10 | 0.006698 | 93 | 0.877966 | 56.10526 | 30.68539 |
| aminoglycoside antibiotic | AAC(6')-Ib7 | 3.914516 | 22 | 0.809524 | 56.10526 | 3911.416 |
| aminoglycoside antibiotic | AAC(6')-Ib8 | 0.006648 | 29 | 0.515152 | 56.10526 | 5.571926 |
| aminoglycoside antibiotic | AAC(6')-Ib9 | 0.003409 | 74 | 0.865672 | 56.10526 | 12.25062 |
| aminoglycoside antibiotic | AAC(6')-Ic | 0.000158 | 15 | 0.85124 | 56.10526 | 0.113471 |
| aminoglycoside antibiotic | AAC(6')-IIa | 0.002476 | 17 | 0.714286 | 56.10526 | 1.686885 |
| aminoglycoside antibiotic | AAC(6')-IIc | 0.000237 | 19 | 0.42 | 56.10526 | 0.106232 |
| aminoglycoside antibiotic | aadA | 0.144339 | 53 | 0.789855 | 56.10526 | 339.0087 |
| aminoglycoside antibiotic | aadA10 | 0.003306 | 8 | 0.571429 | 56.10526 | 0.847837 |
| aminoglycoside antibiotic | aadA12 | 0.028912 | 14 | 1 | 56.10526 | 22.70957 |
| aminoglycoside antibiotic | aadA13 | 0.00948 | 7 | 0.545455 | 56.10526 | 2.03074 |
| aminoglycoside antibiotic | aadA16 | 0.118473 | 40 | 0.733333 | 56.10526 | 194.9774 |
| aminoglycoside antibiotic | aadA17 | 0.032963 | 11 | 0.75 | 56.10526 | 15.25747 |
| aminoglycoside antibiotic | aadA2 | 0.037521 | 155 | 0.895787 | 56.10526 | 292.2925 |
| aminoglycoside antibiotic | aadA22 | 0.068915 | 19 | 0.875 | 56.10526 | 64.28023 |
| aminoglycoside antibiotic | aadA23 | 0.04228 | 46 | 0.966667 | 56.10526 | 105.4797 |
| aminoglycoside antibiotic | aadA24 | 0.119048 | 6 | 0.714286 | 56.10526 | 28.62519 |
| aminoglycoside antibiotic | aadA3 | 0.029078 | 34 | 0.75 | 56.10526 | 41.60182 |
| aminoglycoside antibiotic | aadA4 | 0.006725 | 2 | 0.142857 | 56.10526 | 0.10781 |
| aminoglycoside antibiotic | aadA5 | 0.858646 | 75 | 0.936877 | 56.10526 | 3385.022 |
| aminoglycoside antibiotic | aadA7 | 0.001503 | 10 | 0.785714 | 56.10526 | 0.662766 |
| aminoglycoside antibiotic | aadA8 | 0.037802 | 9 | 1 | 56.10526 | 19.08789 |
| aminoglycoside antibiotic | aadK | 0.000186 | 3 | 0.896739 | 56.10526 | 0.028143 |
| Multidrug | abcA | 2.33E-05 | 7 | 0.183673 | 5261.039 | 0.157501 |
| Multidrug | abeM | 0.004409 | 16 | 0.954936 | 737.8568 | 49.70233 |
| Multidrug | abeS | 0.000637 | 11 | 0.997732 | 870.8962 | 6.090314 |
| Multidrug | acrE | 12.81385 | 57 | 0.965947 | 5987.263 | 4224119 |
| Multidrug | acrF | 12.51105 | 64 | 0.938728 | 5987.263 | 4500303 |
| Beta-lactams | ADC-8 | 0.00449 | 23 | 0.857143 | 1004.408 | 88.90331 |
| Multidrug | adeF | 0.029415 | 608 | 0.371864 | 1425.974 | 9483.415 |
| Multidrug | adeH | 0.002548 | 5 | 0.991914 | 1425.974 | 18.0224 |
| Multidrug | adeI | 0.009383 | 15 | 0.988962 | 3335.059 | 464.2062 |
| aminoglycoside antibiotic | amrA | 0.00068 | 1 | 0.549828 | 56.10526 | 0.02098 |
| aminoglycoside antibiotic | amrB | 0.074948 | 4 | 0.44837 | 56.10526 | 7.54156 |
| aminoglycoside antibiotic | amrB | 0.074948 | 4 | 0.44837 | 56.10526 | 7.54156 |
| aminoglycoside antibiotic | ANT(2'')-Ia | 10.3149 | 81 | 0.789256 | 56.10526 | 36997.43 |
| aminoglycoside antibiotic | ANT(3'')-IIa | 0.488527 | 226 | 0.843157 | 56.10526 | 5222.868 |
| aminoglycoside antibiotic | ANT(3'')-IIc | 0.011276 | 19 | 1 | 56.10526 | 12.02051 |
| aminoglycoside antibiotic | ANT(6)-Ia | 0.400215 | 46 | 0.766129 | 56.10526 | 791.3283 |
| aminoglycoside antibiotic | APH(3')-Ia | 1.288955 | 261 | 0.832955 | 56.10526 | 15721.83 |
| aminoglycoside antibiotic | APH(3')-Ib | 0.091222 | 4 | 0.75 | 56.10526 | 15.35402 |
| aminoglycoside antibiotic | APH(3'')-Ib | 14.87424 | 329 | 0.843956 | 56.10526 | 231714.9 |
| aminoglycoside antibiotic | APH(3')-IIa | 0.062687 | 28 | 0.841584 | 56.10526 | 82.87712 |
| aminoglycoside antibiotic | APH(4)-Ia | 0.005485 | 58 | 0.902041 | 56.10526 | 16.10154 |
| aminoglycoside antibiotic | APH(6)-Ia | 0.000103 | 3 | 0.342593 | 56.10526 | 0.005958 |
| aminoglycoside antibiotic | APH(6)-Ic | 0.41115 | 8 | 0.392857 | 56.10526 | 72.49842 |
| aminoglycoside antibiotic | APH(6)-Id | 16.14434 | 345 | 0.846703 | 56.10526 | 264590.5 |
| peptide antibiotic | arnA | 0.74631 | 6 | 0.968927 | 10.77632 | 46.75537 |
| rifamycin antibiotic | arr-2 | 0.029166 | 37 | 0.920245 | 0.856757 | 0.850821 |
| rifamycin antibiotic | arr-3 | 0.102493 | 89 | 0.83526 | 0.856757 | 6.527743 |
| peptide antibiotic | bacA | 13.11501 | 29 | 0.867508 | 10.77632 | 3555.579 |
| Beta-lactams | BcI | 0.002451 | 5 | 0.469388 | 5250.263 | 30.19848 |
| Beta-lactams | BcII | 0.00435 | 6 | 0.346457 | 5250.263 | 47.47341 |
| peptide antibiotic | bcrA | 0.021417 | 2 | 0.025 | 10.77632 | 0.01154 |
| Beta-lactams | BLA1 | 0.002093 | 1 | 0.870968 | 4245.855 | 7.739668 |
| Beta-lactams | blaF | 0.002417 | 1 | 0.1 | 4245.855 | 1.02625 |
| Multidrug | bmr | 0.000332 | 1 | 0.893939 | 749.041 | 0.222134 |
| phenicol antibiotic | catA8 | 0.150559 | 14 | 0.540541 | 11.18421 | 12.74287 |
| phenicol antibiotic | catB11 | 0.000105 | 13 | 0.533333 | 11.18421 | 0.008142 |
| phenicol antibiotic | catB2 | 0.001329 | 20 | 0.607143 | 11.18421 | 0.180443 |
| phenicol antibiotic | catB3 | 0.01095 | 141 | 0.88225 | 11.18421 | 15.23396 |
| phenicol antibiotic | catB8 | 0.000732 | 27 | 0.941176 | 11.18421 | 0.208096 |
| phenicol antibiotic | catB9 | 0.011722 | 5 | 0.794118 | 11.18421 | 0.520562 |
| phenicol antibiotic | catQ | 0.059988 | 1 | 0.5 | 11.18421 | 0.33546 |
| Multidrug | ceoB | 0.444428 | 12 | 0.211765 | 793.1053 | 895.7087 |
| phenicol antibiotic | cmlA5 | 0.013971 | 56 | 0.930556 | 11.18421 | 8.142532 |
| phenicol antibiotic | cmx | 0.709824 | 28 | 0.44 | 11.18421 | 97.80623 |
| Beta-lactams | CTX-M-3 | 0.011254 | 35 | 0.830189 | 1004.408 | 328.4554 |
| diaminopyrimidine antibiotic | dfrA27 | 0.130866 | 35 | 0.75 | 0.856757 | 2.943162 |
| diaminopyrimidine antibiotic | dfrB4 | 0.005372 | 8 | 0.2 | 0.856757 | 0.007364 |
| diaminopyrimidine antibiotic | dfrD | 0.003746 | 1 | 0.5 | 0.856757 | 0.001605 |
| diaminopyrimidine antibiotic | dfrG | 0.017587 | 32 | 0.845455 | 0.856757 | 0.407649 |
| Multidrug | efrA | 3.052901 | 23 | 0.834123 | 1607.896 | 94173.53 |
| Multidrug | efrB | 3.681096 | 2 | 0.95614 | 1607.896 | 11318.45 |
| fluoroquinolone antibiotic | emrA | 11.61727 | 39 | 0.919714 | 737 | 307106.4 |
| fluoroquinolone antibiotic | emrB | 5.703744 | 42 | 0.851689 | 737 | 150368.8 |
| tetracycline antibiotic | emrK | 10.28333 | 46 | 0.976238 | 688.9737 | 318163 |
| tetracycline antibiotic | emrY | 9.556792 | 63 | 0.968457 | 688.9737 | 401731.4 |
| peptide antibiotic | eptA | 12.51839 | 31 | 0.954034 | 10.77632 | 3989.737 |
| antibacterial free fatty acids | farA | 0.022608 | 3 | 0.98 | 0.856757 | 0.056945 |
| antibacterial free fatty acids | farB | 0.033684 | 9 | 0.9 | 0.856757 | 0.233761 |
| phenicol antibiotic | fexA | 0.006002 | 39 | 0.6 | 11.18421 | 1.570753 |
| phenicol antibiotic | floR | 0.046011 | 155 | 0.843787 | 11.18421 | 67.30232 |
| fosfomycin | FosA3 | 0.016128 | 80 | 0.898618 | 7.866667 | 9.120595 |
| fosfomycin | FosA4 | 0.00342 | 12 | 0.571429 | 7.866667 | 0.184465 |
| fosfomycin | FosB | 0.007844 | 7 | 0.477707 | 7.866667 | 0.20634 |
| fosfomycin | FosC2 | 0.000258 | 2 | 0.75 | 7.866667 | 0.003043 |
| MLS | linG | 0.001057 | 45 | 0.831169 | 0.856757 | 0.033857 |
| Multidrug | lmrC | 0.075301 | 3 | 0.111111 | 1443.785 | 36.2395 |
| Multidrug | lmrP | 0.002561 | 7 | 0.985019 | 1427.687 | 25.21374 |
| MLS | LpeB | 0.000145 | 1 | 0.75 | 870.0395 | 0.094427 |
| MLS | macA | 0.293242 | 3 | 0.942857 | 870.0395 | 721.6596 |
| MLS | macB | 1.441188 | 12 | 0.929907 | 870.0395 | 13992.01 |
| Multidrug | mdsB | 0.087717 | 7 | 0.978982 | 5283.296 | 3175.856 |
| Multidrug | mdsC | 0.001004 | 11 | 0.99798 | 5283.296 | 58.21669 |
| Multidrug | mdtE | 13.49905 | 38 | 0.96396 | 5852.895 | 2894119 |
| Multidrug | mdtF | 13.09669 | 40 | 0.936249 | 5852.895 | 2870674 |
| fluoroquinolone antibiotic | mdtH | 9.373798 | 17 | 0.962282 | 737 | 113014.5 |
| fluoroquinolone antibiotic | mdtK | 0.793789 | 29 | 0.994995 | 737 | 16880.73 |
| Multidrug | mdtN | 10.79679 | 25 | 0.959643 | 0.856757 | 221.9229 |
| Multidrug | mel | 8.722298 | 9 | 0.578947 | 1443.785 | 65616.81 |
| Multidrug | MexA | 0.34145 | 3 | 0.994236 | 7592.656 | 7732.719 |
| Multidrug | MexB | 1.698301 | 3 | 0.991643 | 7592.656 | 38360.59 |
| Multidrug | MexC | 0.084871 | 3 | 0.985755 | 7615.279 | 1911.333 |
| Multidrug | MexD | 0.66404 | 4 | 0.966387 | 7615.279 | 19547.5 |
| Multidrug | MexE | 0.680515 | 6 | 0.97971 | 749.041 | 2996.349 |
| Multidrug | MexF | 2.46713 | 3 | 0.994236 | 749.041 | 5511.991 |
| Multidrug | mexG | 0.013941 | 5 | 0.997076 | 1425.974 | 99.10434 |
| Multidrug | mexJ | 0.087052 | 1 | 0.988636 | 1559.87 | 134.2461 |
| Multidrug | mexK | 1.387887 | 4 | 0.947945 | 1559.87 | 8208.915 |
| phenicol antibiotic | mexN | 0.37918 | 3 | 0.982709 | 11.18421 | 12.5025 |
| Multidrug | mexP | 0.010696 | 1 | 0.994118 | 1591.08 | 16.91771 |
| Multidrug | mexQ | 0.197569 | 6 | 0.96338 | 1591.08 | 1817.021 |
| Multidrug | mexW | 0.538899 | 4 | 0.92328 | 2307.197 | 4591.826 |
| nitroimidazole antibiotic | msbA | 11.77791 | 58 | 0.779501 | 0.856757 | 456.2156 |
| Multidrug | mtrD | 0.248121 | 11 | 0.932692 | 5115.895 | 13023.14 |
| Multidrug | mtrE | 0.081651 | 2 | 0.847059 | 5115.895 | 707.6651 |
| MLS | myrA | 0.006097 | 3 | 0.058824 | 737.8568 | 0.793871 |
| Multidrug | norA | 2.204777 | 2 | 0.91 | 737 | 2957.355 |
| Multidrug | OpmB | 0.439013 | 9 | 0.992021 | 1560.835 | 6117.844 |
| Multidrug | opmE | 0.005451 | 10 | 0.974063 | 1591.08 | 84.47373 |
| triclosan | OpmH | 0.831499 | 1 | 0.961003 | 0.856757 | 0.684612 |
| Multidrug | oprA | 0.204448 | 3 | 0.2 | 2372.145 | 290.9884 |
| Multidrug | OprJ | 0.031707 | 2 | 0.97971 | 7615.279 | 473.1157 |
| Multidrug | OprM | 0.6207 | 3 | 0.977465 | 7648.761 | 13921.8 |
| Multidrug | OprN | 0.492287 | 1 | 0.985465 | 749.041 | 363.3835 |
| Multidrug | optrA | 0.010647 | 25 | 0.666667 | 1443.785 | 256.1969 |
| Multidrug | oqxA | 0.657784 | 101 | 0.656498 | 1428.054 | 62284.93 |
| Multidrug | oqxB | 1.2047 | 103 | 0.701453 | 1428.054 | 124296.6 |
| Beta-lactams | OXA-2 | 0.000144 | 29 | 0.511111 | 5270.289 | 11.23955 |
| Beta-lactams | OXA-21 | 0.002108 | 9 | 0.714286 | 5250.263 | 71.13969 |
| Beta-lactams | OXA-309 | 0.00116 | 1 | 1 | 5250.263 | 6.089289 |
| Beta-lactams | OXA-444 | 0.001831 | 1 | 0.8 | 5250.263 | 7.688962 |
| Beta-lactams | OXA-486 | 0.004907 | 2 | 1 | 5250.263 | 51.52128 |
| Beta-lactams | OXA-50 | 0.002047 | 7 | 0.808511 | 5250.263 | 60.82244 |
| fluoroquinolone antibiotic | patA | 0.752058 | 20 | 0.987805 | 737 | 10950.15 |
| Multidrug | PmpM | 0.439384 | 5 | 0.927152 | 793.962 | 1617.206 |
| fluoroquinolone antibiotic | pmrA | 0.490293 | 2 | 0.978723 | 737 | 707.3154 |
| peptide antibiotic | pmrF | 10.90395 | 39 | 0.865254 | 10.77632 | 3965.174 |
| phenicol antibiotic | pp-flo | 0.012163 | 7 | 0.25 | 11.18421 | 0.23806 |
| fluoroquinolone antibiotic | qacH | 0.012668 | 7 | 0.428571 | 737 | 28.0094 |
| fluoroquinolone antibiotic | QepA2 | 0.006925 | 10 | 0.923077 | 737 | 47.10988 |
| fluoroquinolone antibiotic | QepA4 | 0.008543 | 7 | 0.8 | 737 | 35.25944 |
| peptide antibiotic | rosB | 0.017622 | 1 | 0.090909 | 10.77632 | 0.017264 |
| rifamycin antibiotic | rphB | 0.000468 | 4 | 0.166667 | 0.856757 | 0.000267 |
| Multidrug | smeD | 0.453553 | 5 | 0.820513 | 2307.197 | 4293.071 |
| Multidrug | smeE | 1.203099 | 3 | 0.788235 | 2307.197 | 6563.919 |
| Multidrug | smeF | 0.484148 | 3 | 0.84507 | 2307.197 | 2831.894 |
| sulfonamide antibiotic | sul1 | 10.59813 | 301 | 0.852966 | 0.856757 | 2331.23 |
| sulfonamide antibiotic | sul2 | 7.477222 | 271 | 0.854417 | 0.856757 | 1483.327 |
| sulfonamide antibiotic | sul4 | 0.002379 | 5 | 0.5 | 0.856757 | 0.005096 |
| Diterpenoids | TaeA | 0.367144 | 2 | 0.4 | 0.856757 | 0.251642 |
| tetracycline antibiotic | tap | 0.005266 | 2 | 0.222222 | 688.9737 | 1.612643 |
| Beta-lactams | TEM-1 | 0.103428 | 319 | 0.900277 | 1005.373 | 29863.04 |
| tetracycline antibiotic | tet(30) | 0.000101 | 2 | 0.142857 | 688.9737 | 0.01979 |
| tetracycline antibiotic | tet(39) | 0.004115 | 17 | 0.617647 | 688.9737 | 29.76931 |
| tetracycline antibiotic | tet(40) | 23.54252 | 4 | 0.615385 | 688.9737 | 39926.6 |
| tetracycline antibiotic | tet(41) | 0.000374 | 2 | 0.83 | 688.9737 | 0.427176 |
| tetracycline antibiotic | tet(42) | 0.22004 | 5 | 0.375 | 688.9737 | 284.2527 |
| tetracycline antibiotic | tet(A) | 1.488588 | 161 | 0.895077 | 688.9737 | 147796.2 |
| tetracycline antibiotic | tet(B) | 3.735535 | 141 | 0.860668 | 688.9737 | 312327.4 |
| tetracycline antibiotic | tet(C) | 0.193933 | 51 | 0.617391 | 688.9737 | 4207.123 |
| tetracycline antibiotic | tet(G) | 0.004712 | 1 | 0.090909 | 688.9737 | 0.295111 |
| tetracycline antibiotic | tet(L) | 0.839716 | 48 | 0.460123 | 688.9737 | 12777.61 |
| tetracycline antibiotic | Tet(X4) | 2.54922 | 27 | 0.708861 | 690.1976 | 33674.83 |
| tetracycline antibiotic | tetA(60) | 0.088639 | 1 | 0.363636 | 688.9737 | 22.20716 |
| tetracycline antibiotic | tetA(P) | 0.518035 | 3 | 0.757576 | 688.9737 | 811.164 |
| tetracycline antibiotic | tetB(60) | 0.096513 | 2 | 0.2 | 688.9737 | 26.59786 |
| tetracycline antibiotic | tetB(P) | 0.225755 | 10 | 0.042169 | 688.9737 | 65.58892 |
| Multidrug | tolC | 13.60416 | 24 | 0.940823 | 7649.019 | 2349615 |
| peptide antibiotic | ugd | 9.03761 | 91 | 0.963171 | 10.77632 | 8536.279 |
| glycopeptide antibiotic | vanB | 0.034679 | 8 | 0.9 | 8.776316 | 2.191332 |
| glycopeptide antibiotic | vanG | 0.236757 | 3 | 0.059701 | 8.776316 | 0.372153 |
| glycopeptide antibiotic | vanHB | 0.034351 | 9 | 0.931034 | 8.776316 | 2.526143 |
| glycopeptide antibiotic | vanI | 0.10015 | 15 | 0.018293 | 8.776316 | 0.241174 |
| glycopeptide antibiotic | vanM | 0.000844 | 4 | 0.5 | 8.776316 | 0.014822 |
| glycopeptide antibiotic | vanXA | 0.002198 | 32 | 0.950355 | 8.776316 | 0.58666 |
| glycopeptide antibiotic | vanXB | 0.036752 | 7 | 0.875 | 8.776316 | 1.975606 |
| glycopeptide antibiotic | vanXD | 0.08754 | 1 | 0.5 | 8.776316 | 0.384141 |
| glycopeptide antibiotic | vanXI | 0.000499 | 3 | 0.461538 | 8.776316 | 0.006066 |
| glycopeptide antibiotic | vanXO | 9.31E-05 | 3 | 0.333333 | 8.776316 | 0.000817 |
| glycopeptide antibiotic | vanYA | 0.006229 | 28 | 0.954198 | 8.776316 | 1.460558 |
| glycopeptide antibiotic | vanZA | 0.00049 | 26 | 0.961832 | 8.776316 | 0.107599 |
| Multidrug | ykkD | 0.00016 | 2 | 0.875622 | 756.2632 | 0.211357 |

These data used to calculate the risk index for each ARG were obtained from the dataset established by Zhang et al [1].

**Table S6. Details information of 76 high-quality** **MAGs from phenotypic resistance communities detected in this study.**

| MAGID | Classification | Number of ARGs | Number of multidrug resistant genes | Number of quinolone resistant genes | Cd resistant genes |  |
| --- | --- | --- | --- | --- | --- | --- |
| S12C2969 | g__UBA4001 | 128 | 20 | 0 | *czcD, czcR* |  |
| S13C2969 | g__UBA4001 | 128 | 20 | 0 | *czcD, czcR* |  |
| S14C2969 | g__UBA4001 | 125 | 19 | 0 | *czcD, czcR* |  |
| S15C2969 | g__UBA4001 | 121 | 20 | 0 | *czcD, czcR* |  |
| S1C2969 | g__UBA4001 | 128 | 20 | 0 | *czcD, czcR* |  |
| S22C2969 | g__UBA4001 | 126 | 20 | 0 | *czcD, czcR* |  |
| S24C2969 | g__UBA4001 | 122 | 20 | 0 | *czcD, czcR* |  |
| S23C2969 | g__UBA4001 | 124 | 20 | 0 | *czcD, czcR* |  |
| S5C2969 | g__UBA4001 | 126 | 20 | 0 | *czcD, czcR* |  |
| S12C157 | s__Sphingopyxis sp000756385 | 125 | 40 | 1 | *czcP* |  |
| S13C157 | s__Sphingopyxis sp000756385 | 123 | 40 | 1 | *czcP* |  |
| S14C157 | s__Sphingopyxis sp000756385 | 123 | 40 | 1 | *czcP* |  |
| S15C157 | s__Sphingopyxis sp000756385 | 123 | 40 | 1 | *czcP* |  |
| S16C157 | s__Sphingopyxis sp000756385 | 109 | 35 | 1 | *czcP* |  |
| S17C157 | s__Sphingopyxis sp000756385 | 120 | 40 | 1 | *czcP* |  |
| S18C157 | s__Sphingopyxis sp000756385 | 112 | 39 | 0 | *czcP* |  |
| S1C157 | s__Sphingopyxis sp000756385 | 120 | 40 | 1 | *czcP* |  |
| S22C157 | s__Sphingopyxis sp000756385 | 123 | 40 | 1 | *czcP* |  |
| S23C157 | s__Sphingopyxis sp000756385 | 123 | 40 | 1 | *czcP* |  |
| S24C157 | s__Sphingopyxis sp000756385 | 123 | 40 | 1 | *czcP* |  |
| S31C157 | s__Sphingopyxis sp000756385 | 116 | 35 | 1 | *czcP* |  |
| S5C157 | s__Sphingopyxis sp000756385 | 121 | 40 | 1 | *czcP* |  |
| S32C157 | s__Sphingopyxis sp000756385 | 105 | 30 | 1 | *czcP* |  |
| S33C157 | s__Sphingopyxis sp000756385 | 122 | 39 | 1 | *czcP* |  |
| S12C209 | g__172606-1 | 239 | 48 | 1 | *czcA, czcR* |  |
| S13C209 | g__172606-1 | 240 | 48 | 1 | *czcA, czcR* |  |
| S14C209 | g__172606-1 | 238 | 48 | 1 | *czcA, czcR* |  |
| S15C209 | g__172606-1 | 238 | 48 | 1 | *czcA, czcR* |  |
| S1C209 | g__172606-1 | 239 | 48 | 1 | *czcA, czcR* |  |
| S22C209 | g__172606-1 | 234 | 48 | 1 | *czcA, czcR* |  |
| S5C1042 | g__Flavisolibacter | 158 | 38 | 0 | *czcA, czcD, czcP* |  |
| S5C209 | g__172606-1 | 240 | 48 | 1 | *czcA, czcR* |  |
| S33C214 | g__Paenibacillus_AE | 236 | 47 | 0 | *cadD, czcP* |  |
| S10C696 | f__Thermithiobacillaceae | 98 | 20 | 0 | *cadR, czcA, cznA* |  |
| S12C696 | f__Thermithiobacillaceae | 98 | 20 | 0 | *cadR, czcA, cznA* |  |
| S13C696 | f__Thermithiobacillaceae | 95 | 17 | 0 | *cadR, czcA, cznA* |  |
| S14C60 | g__CABHOJ01 | 80 | 15 | 0 | *czcA* |  |
| S14C696 | f__Thermithiobacillaceae | 98 | 20 | 0 | *cadR, czcA, cznA* |  |
| S1C696 | f__Thermithiobacillaceae | 98 | 20 | 0 | *cadR, czcA, cznA* |  |
| S22C696 | f__Thermithiobacillaceae | 97 | 20 | 0 | *cadR, czcA, cznA* |  |
| S23C696 | f__Thermithiobacillaceae | 97 | 20 | 0 | *cadR, czcA, cznA* |  |
| S24C696 | f__Thermithiobacillaceae | 97 | 20 | 0 | *cadR, czcA, cznA* |  |
| S26C696 | f__Thermithiobacillaceae | 94 | 17 | 0 | *cadR, czcA, cznA* |  |
| S27C696 | f__Thermithiobacillaceae | 97 | 20 | 0 | *cadR, czcA, cznA* |  |
| S29C696 | f__Thermithiobacillaceae | 94 | 17 | 0 | *cadR, czcA, cznA* |  |
| S30C696 | f__Thermithiobacillaceae | 97 | 20 | 0 | *cadR, czcA, cznA* |  |
| S11C4952 | s__Agrococcus lahaulensis_A | 95 | 12 | 2 | *cadD, czcD* |  |
| S28C4952 | s__Agrococcus lahaulensis_A | 94 | 12 | 2 | *cadD, czcD* |  |
| S26C4952 | s__Agrococcus lahaulensis_A | 90 | 12 | 2 | *czcD* |  |
| S23C209 | g__172606-1 | 236 | 48 | 1 | *czcA, czcR* |  |
| S24C209 | g__172606-1 | 237 | 47 | 1 | *czcA, czcR* |  |
| S12C60 | g__CABHOJ01 | 89 | 19 | 0 | *czcA, czcD, czrB* |  |
| S13C60 | g__CABHOJ01 | 74 | 13 | 0 | *czcA* |  |
| S1C60 | g__CABHOJ01 | 88 | 18 | 0 | *czcA, czcD, czrB* |  |
| S22C60 | g__CABHOJ01 | 88 | 18 | 0 | *czcA, czcD, czrB* |  |
| S23C60 | g__CABHOJ01 | 89 | 19 | 0 | *czcA, czcD, czrB* |  |
| S24C60 | g__CABHOJ01 | 89 | 19 | 0 | *czcA, czcD, czrB* |  |
| S25C60 | g__CABHOJ01 | 75 | 14 | 0 | *czcA, cznA* |  |
| S26C60 | g__CABHOJ01 | 81 | 18 | 0 | *czcA, czcD, czrB* |  |
| S27C60 | g__CABHOJ01 | 85 | 18 | 0 | *czcA, czcD, cznA, czrB* |  |
| S5C60 | g__CABHOJ01 | 90 | 20 | 0 | *czcA, czcD, czrB* |  |
| S16C363 | g__Devosia | 250 | 51 | 1 | *czcP* |  |
| S17C363 | g__Devosia | 248 | 51 | 1 | *czcP* |  |
| S18C363 | g__Devosia | 249 | 52 | 1 | *czcP* |  |
| S19C363 | g__Devosia | 247 | 51 | 1 | *czcP* |  |
| S20C363 | g__Devosia | 250 | 51 | 1 | *czcP* |  |
| S21C363 | g__Devosia | 248 | 51 | 1 | *czcP* |  |
| S33C363 | g__Devosia | 232 | 48 | 1 | *czcD* |  |
| S12C177 | g__Flavisolibacter | 149 | 31 | 0 | *cadD, czcA, czcD* |  |
| S13C177 | g__Flavisolibacter | 149 | 31 | 0 | *cadD, czcA, czcD* |  |
| S14C177 | g__Flavisolibacter | 149 | 31 | 0 | *cadD, czcA, czcD* |  |
| S15C177 | g__Flavisolibacter | 149 | 31 | 0 | *cadD, czcA, czcD* |  |
| S1C177 | g__Flavisolibacter | 149 | 31 | 0 | *cadD, czcA, czcD* |  |
| S30C89 | g__Fluviicola | 113 | 31 | 0 | *czcA, czcD* |  |
| S33C107 | s__Flavisolibacter sp013327885 | 162 | 35 | 2 | *czcA, czcD, czcP* |  |
| S5C177 | g__Flavisolibacter | 149 | 31 | 0 | *cadD, czcA, czcD* |  |

**Table S7. PCR primer sequences for multidrug resistant genes validation.**

| Target gene | Forward sequence (5'-3') | Reverse sequence (5'-3') |
| --- | --- | --- |
| *mdtA* | ACAAGCCCAGGGCCAAC | CCTTAATGGTGCCTTCGGTTTC |
| *acrB* | AGTCGGTGTTCGCCGTTAAC | CAAGGAAACGAACGCAATACC |
| *mexA* | AGGACAACGCTATGCAACGAA | CCGGAAAGGGCCGAAAT |
| *mexB* | CTGGAGATCGACGACGAGAAG | GAAATCGTTGACGTAGCTGGAA |

**Table S8. Genomic co-localization of ARGs, CdRGs and MGEs (mobile genetic elements) on contigs within MAGs from phenotypic resistance communities.**

| MAG | contig in MAG | length | ARG | ARG_start | ARG_end | ARG_+/- | CdRG | CdRG_start | CdRG_end | CdRG_+/- | distance | MGE_ subtype | MGE_start | MGE_end | MGE_+/- |
| --- | --- | --- | --- | --- | --- | --- | --- | --- | --- | --- | --- | --- | --- | --- | --- |
| S1C60 | gnl\|S1C60\|S1C60_16 | 184826 | *multidrug_ABC_transporter* | 171145 | 172983 | - | *czcA* | 174727 | 177822 | - | 1744 | *tniA* | 184228 | 184545 | - |
| S12C60 | gnl\|S12C60\|S12C60_7 | 184826 | *multidrug_ABC_transporter* | 171145 | 172983 | - | *czcA* | 174727 | 177822 | - | 1744 | *tniA* | 184228 | 184545 | - |

**Table S9. Changes in soil NH₄⁺ and available phosphorous concentration during the remediation process.**

| Concentration  (mg kg^-1^) | L 0 day | L 60 day | L 120 day | M 0 day | M 60 day | M 120 day | H 0 day | H 60 day | H 120 day |
| --- | --- | --- | --- | --- | --- | --- | --- | --- | --- |
| NH4+ | 15.68$\pm0.04$ | 18.30$\pm0.63$ | 19.22$\pm0.68$ | 15.63$\pm0.65$ | 18.54$\pm1.04$ | 20.00$\pm1.17$ | 15.81$\pm0.35$ | 18.81$\pm1.68$ | 18.80$\pm2.70$ |
| Available phosphorous | 12.15$\pm0.35$ | 11.44$\pm0.31$ | 9.88$\pm0.36$ | 11.89$\pm0.29$ | 13.52$\pm0.53$ | 11.64$\pm0.93$ | 15.35$\pm0.25$ | 17.09$\pm0.33$ | 14.17$\pm0.28$ |

**References**

1. Zhang Z, Zhang Q, Wang T *et al.* Assessment of global health risk of antibiotic resistance genes. *Nat Commun*. 2022;**13**:1553 <https://doi.org/10.1038/s41467-022-29283-8>
